# Cognitive simulation along with neural adaptation explain effects of suggestions: a novel theoretical framework

**DOI:** 10.3389/fpsyg.2024.1388347

**Published:** 2024-06-20

**Authors:** Anoushiravan Zahedi, Steven Jay Lynn, Werner Sommer

**Affiliations:** ^1^Department of Psychology, University of Münster, Münster, Germany; ^2^Department of Psychology, Humboldt-Universitaet zu Berlin, Berlin, Germany; ^3^Neuroscience Research Center, Charité-Universitätsmedizin Berlin, Berlin, Germany; ^4^Department of Psychology, Binghamton University, Binghamton, NY, United States; ^5^Department of Psychology, Zhejiang Normal University, Jinhua, China; ^6^Department of Physics and Life Science Imaging Center, Hong Kong Baptist University, Kowloon, Hong Kong SAR, China; ^7^Faculty of Education, National University of Malaysia, Kuala Lumpur, Malaysia

**Keywords:** hypnotic suggestions, hypnosis, suggestibility, cognitive simulation, neural adaptation, sensory attenuation, learning, predictive coding model

## Abstract

Hypnosis is an effective intervention with proven efficacy that is employed in clinical settings and for investigating various cognitive processes. Despite their practical success, no consensus exists regarding the mechanisms underlying well-established hypnotic phenomena. Here, we suggest a new framework called the Simulation-Adaptation Theory of Hypnosis (SATH). SATH expands the predictive coding framework by focusing on (a) redundancy elimination in generative models using intrinsically generated prediction errors, (b) adaptation due to amplified or prolonged neural activity, and (c) using internally generated predictions as a venue for learning new associations. The core of our treatise is that simulating proprioceptive, interoceptive, and exteroceptive signals, along with the top-down attenuation of the precision of sensory prediction errors due to neural adaptation, can explain objective and subjective hypnotic phenomena. Based on these postulations, we offer mechanistic explanations for critical categories of direct verbal suggestions, including (1) direct-ideomotor, (2) challenge-ideomotor, (3) perceptual, and (4) cognitive suggestions. Notably, we argue that besides explaining objective responses, SATH accounts for the subjective effects of suggestions, i.e., the change in the sense of agency and reality. Finally, we discuss individual differences in hypnotizability and how SATH accommodates them. We believe that SATH is exhaustive and parsimonious in its scope, can explain a wide range of hypnotic phenomena without contradiction, and provides a host of testable predictions for future research.

## 1 Introduction

Hypnosis is an effective intervention used in clinical settings, either as a stand-alone or an adjunct to other methods and techniques, such as cognitive-behavior therapy (e.g., Schoenberger, 2000), among others, in treating depression (e.g., Alladin and Alibhai, 2007), anxiety-related disorders (e.g., Valentine et al., 2019), acute and chronic pain (e.g., Thompson et al., 2019), obesity and overweight (e.g., Kirsch, 1996; Milling et al., 2018), and enhancing self-acceptance (e.g., Milburn, 2010). In basic and applied psychological research, hypnotic and posthypnotic suggestions are frequently employed to enhance psychological functions and investigate their neurocognitive underpinnings, such as inhibition (e.g., Iani et al., 2006; Raz et al., 2006; Augustinova and Ferrand, 2012; Zahedi et al., 2017, 2019), working memory (e.g., Lindelov et al., 2017; Zahedi et al., 2020b), perception (e.g., Derbyshire et al., 2004; McGeown et al., 2012; Perri et al., 2019), and implicit motivation (e.g., Ludwig et al., 2014; Zahedi et al., 2020a). Accordingly, hypnosis is an established procedure with proven efficacy.

However, as highlighted by several reviews (Sheehan and Perry, 1976; Lynn and Rhue, 1991; Zahedi et al., 2021), there is no consensus about the mechanisms underlying the effects of hypnosis and hypnotic suggestions. What is common to the phenomena subsumed under the name of hypnosis, and why are hypnotic suggestions effective in changing such a diverse array of functions ranging from behavior, perception, and cognition to the subjective sense of agency (SoA) and the sense of reality (SoR)? To address these questions, we propose a new theory of hypnosis, which, based on criteria outlined by philosophers of science, such as Popper (1971), (I) accounts for as many phenomena as possible without contradiction (i.e., adequacy) and (II) makes as few assumptions as possible (i.e., parsimony). Notably, this new theory intends to incorporate previous theories [for a systematic review, see Zahedi et al., 2021] and, hence, adopts many of their principles and elements. In the following, we will (I) briefly introduce hypnosis and hypnotic phenomena and (II) discuss the predictive coding framework (PCF) as the basis of understanding action, perception, and cognition (Friston, 2010; Clark, 2013). (III) Finally, we will propose our new framework, the simulation-adaptation theory of hypnosis (SATH), which is based on the PCF and can parsimoniously explain a wide range of hypnotic phenomena without internal contradiction.

## 2 Hypnosis and hypnotic phenomena

Hypnosis is best described as a procedure that consists of at least three separated phases (Hammond, 1998; Kihlstrom, 2008), namely, induction, an intermediary stage that includes various hypnotic and/or posthypnotic suggestions, and termination (also called de-induction). All three stages are induced in the participant via verbal suggestions that another person, called the hypnotist, presents (Kihlstrom, 1985; Lynn et al., 2015a,b). The suggestions employed are direct verbal suggestions and aim to build a suggested reality that might contradict the actual reality as it is experienced and known by the hypnotized participant (Polczyk, 2016; Oakley et al., 2021).

Although relaxation suggestions are commonly used for the induction phase (Edmonston, 1977, 1991), it is well-established that hypnosis can be induced even during strenuous physical activity (Banyai and Hilgard, 1976; Malott, 1984). Further, previous studies have hinted that hypnotic induction might have little or no effect on participants’ responsiveness (Mazzoni et al., 2009; McGeown et al., 2012) and may not be necessary for the effectiveness of direct verbal suggestions (Parris and Dienes, 2013). Hence, although an induction phase is part of the standard hypnotic procedure, its contribution to the efficacy of the following direct verbal suggestions is not well established (Braffman and Kirsch, 1999; Lynn et al., 2015b). Therefore, in the following, we will focus on the effects of direct verbal suggestions, regardless of the presence or type of induction phase employed.

One may categorize direct verbal suggestions based on their content. For instance, Hilgard (1965a), proposed that suggestions can be divided into (I) agnosia and cognitive distortion, (II) positive hallucinations, (III) negative hallucinations, (IV) dreams and regressions, (V) amnesia and posthypnotic suggestion, and finally, (VI) loss of motor control. However, attempts to categorize suggestions using factor analyses have resulted in a different picture (Hilgard, 1965a,b; McConkey et al., 1980; Woody et al., 2005). Most analyses (McConkey et al., 1980; Woody et al., 2005) yielded at least three factors, commonly termed direct-ideomotor, challenge-ideomotor, and perceptual-cognitive (McConkey et al., 1980; Woody et al., 2005). These terms were introduced in the 1940ies by Eysenck (1943) and Eysenck and Furneaux (1945), who investigated general suggestibility and its relationship with hypnotizability; however, the definition of the terms changed thereafter.

Direct-ideomotor suggestions usually induce a movement in the participant by suggesting to think about a movement itself or its known precursors. For instance, the following suggestion from the Harvard group scale of hypnotic susceptibility (HGSHS-A; 46, 47) is considered a standard example of direct-ideomotor suggestions: “Please hold both hands up in the air. I want you to imagine a force attracting your hands toward each other, pulling them together. As you think of this force pulling your hands together, they will move together” (p. 9). The participant is considered to be objectively responsive if their hands noticeably move toward each other. Challenge-ideomotor items, on the other hand, aim to inhibit a motor response despite a secondary suggestion to override the primary suggestion. Consider, for instance, the “finger interlock” suggestion of the HGSHS-A: “Interlock your fingers and press your hands tightly together. Notice how your fingers are so tightly interlocked together that you wonder very much if you could take your fingers and hands apart. I want you to try to take your hands apart” (Shor and Orne, 1962). Here, the participant is assumed to be objectively responsive if their hands remain interlocked.

In contrast to direct-ideomotor and challenge-ideomotor suggestions, perceptual-cognitive suggestions are less well-delineated. As the term indicates, perceptual-cognitive suggestions attempt to alter the perception or a cognitive process. Common perceptual suggestions are positive and negative hallucinations; both categories try to build an altered reality where either a real object cannot be perceived (i.e., negative hallucination) or an imaginary one is perceived (i.e., positive hallucination). For instance, a typical positive hallucination is a suggestion to see a grayscale image in colors (Mazzoni et al., 2009; McGeown et al., 2012), while commonly used negative hallucinations are hypnosis-induced pain reductions (for review, see Perri et al., 2019, 2020; Thompson et al., 2019).

Three well-established and investigated cognitive effects of direct verbal suggestions will be discussed briefly next: (A) Posthypnotic amnesia occurs when, in response to a direct verbal suggestion, the participant forgets the events that happened during hypnosis after its termination (Kihlstrom, 1980; Kihlstrom, 2014). Posthypnotic amnesia is related to source amnesia rather than episodic memory and pertains to modulations of explicit but not implicit memory (Bryant et al., 1999; David et al., 2000; Barnier et al., 2001; Kihlstrom, 2014). (B) Direct verbal suggestions can enhance several executive functions, a group of cognitive abilities required when responding to a novel task and/or situation (Miyake et al., 2000; Diamond, 2013). For instance, direct verbal posthypnotic suggestions can enhance cognitive control, as required by the Stroop (Raz et al., 2006; Parris and Dienes, 2013; Zahedi et al., 2019), Simon (Iani et al., 2009), flanker (Iani et al., 2006), and Go-NoGo (Zahedi et al., 2020a) tasks. Further, posthypnotic suggestions can boost working memory (Lindelov et al., 2017; Zahedi et al., 2020b). Notably, the effects of direct verbal suggestions cannot be attributed to alterations in bottom-up processes but are related to top-down processes (Terhune et al., 2017; Zahedi et al., 2020b). This conclusion was derived based on two sets of results. First, previous studies showed that direct verbal suggestions can affect performance in tasks where disrupting bottom-up processes is detrimental rather than beneficial, such as the working memory index (Lindelov et al., 2017) and the tone monitoring task (Zahedi et al., 2020b). Second, in tasks where both disruption of bottom-up processes and improving top-down modulations can enhance performance (e.g., in the Stroop, Simon, and Flanker tasks), participants rely heavily on proactive cognitive control when suggestions are active, as measured by EEG band frequencies (Zahedi et al., 2017), event-related potentials (Zahedi et al., 2019), and pupillometry (Parris et al., 2021). Finally, (C) direct verbal suggestions can affect value-based decision-making via shifting preferences (Ludwig et al., 2014; Zahedi et al., 2020a, 2023). For instance, by inducing preferences for healthy food items, posthypnotic suggestions can shift participants’ choices toward healthy food and promote faster rejection of unhealthy items (Ludwig et al., 2014; Zahedi et al., 2020a, 2023).

Responses to direct verbal suggestions would not be considered unique if it were not for the altered SoA and SoR that accompany these responses (Kihlstrom, 2008; Lynn et al., 2015b; Martin and Pacherie, 2019). The altered SoA refers to reports of automaticity, effortlessness, and involuntariness when responding to direct verbal suggestions (Lynn et al., 1990; Kirsch and Lynn, 1997; Blakemore et al., 2003). Additionally, direct verbal suggestions affect how participants perceive their surroundings and themselves in that environment (Kihlstrom, 2008), which is referred to as the SoR.

Two points need to be considered when discussing the SoA and SoR. (I) The SoA can itself be divided into two factors: effortlessness and involuntariness (Polito et al., 2013). Although involuntariness is stable across different settings, effortlessness is more volatile and dependent on other variables and antecedents (Polito et al., 2013). (II) There is a strong association between the experience of involuntariness and responsiveness to direct verbal suggestions (Bowers et al., 1988; Polito et al., 2013). Furthermore, the altered SoR is essential for dissociating hypnotic from non-hypnotic suggestions (Spanos and Barber, 1968).

As a caveat, in the description of the hypnotic phenomena above, we used somewhat deterministic terms and notions. In reality, however, direct verbal suggestions are not as clean-cut as described. To understand this point, one can consider the results of Woody et al. (2005), who used factor analyses to categorize direct verbal suggestions in the HGSHS-A and Stanford hypnotic susceptibility scale (SHSS-C; 67). Based on their results, they concluded that “the perceptual-cognitive items in the HGSHS-A (fly hallucination and posthypnotic suggestion) behaved like direct motor items, whereas the motor challenge items in the SHSS-C (arm rigidity and arm immobilization) behaved like perceptual-cognitive items” (42[p. 210]).

In the next section, we will discuss the PCF and its critical elements required for explaining action, perception, and cognition in a unitary framework.

## 3 The predictive coding framework

As its foundation, SATH relies on the PCF (Friston, 2010; Clark, 2013; Yon et al., 2019). The PCF,^1^ which can be traced back to Helmholtz’s propositions, suggests that the brain acts like a scientist trying to model the world, considering its uncertainties, instead of being merely a passive receiver of external information (Clark, 2013). The currently popular version of predictive coding (Friston, 2010; Adams et al., 2013; Brown et al., 2013) assumes that the brain uses Bayesian-type modeling, constituted of three integral elements: priors (i.e., epistemological uncertainty), evidence, and posteriors (i.e., updated epistemological uncertainty). Empirical priors are top-down predictions (i.e., efferent signals) based on the agent’s generative or heuristic models (Clark, 2013). These predictions constantly interact with exteroceptive (including somatosensory), proprioceptive, and interoceptive evidence (i.e., afferent signals). When predictions cannot account for the evidence, there will be residual epistemological uncertainty, called prediction errors (Friston, 2010). In the short term, prediction errors indicate a “newsworthy” event and enforce perceptual inference (Clark, 2013; Barron et al., 2020). In the long term, prediction errors underwrite learning, where the agent updates empirical priors by considering the probability of priors given the evidence. This process results in the generation of more accurate (c.f., adequate) predictions for the next time around (Clark, 2013).

Two critical elements for applying the PCF to hypnosis are (A) hierarchical organization and (B) precision weighting (Friston, 2010; Clark, 2013). (A) Predictions are organized hierarchically, meaning each neural layer propagates predictions downward and prediction errors upward. In other words, the generative model of each neural layer forms priors required for the predictions of the next level. On the other hand, the prediction error at each level is formed as part of the incoming signal (i.e., the prediction error at the lower level) that could not be accounted for using predictions of the current level. Hence, each level needs only to “explain away” the part of the information that lower levels could not explain away and to send the part that remains to be explained upward (Friston, 2010; Yon et al., 2019). Consequently, the high-level predictions are abstract and amodal, and as the predictions go down the hierarchy, they become more concrete, specific, and modal (Beni, 2022). Further, although at higher levels, predictions are stable and related to our beliefs and goals, at lower levels, they become sensory-oriented and need to be changed at higher frequency rates to keep up with the sensory information (Clark, 2013; Jones and Wilkinson, 2020). (B) Not all predictions can be precise; therefore, the brain makes second-level predictions about the precision of its predictions and prediction errors (Yon et al., 2019). The relative precision of predictions and prediction errors determines whether prediction errors are “newsworthy” and, hence, should be attended to or irrelevant and can be ignored (Auksztulewicz and Friston, 2015).

**Figure 1:**
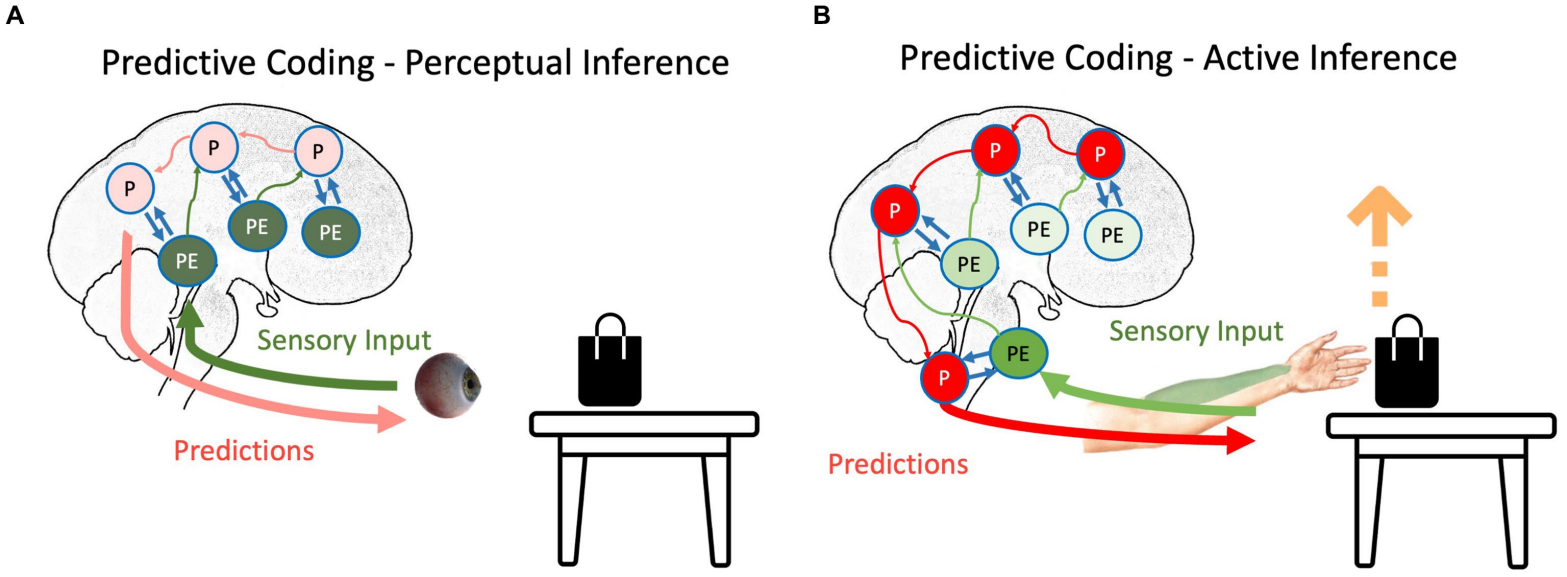
The schematic representation of **(A)** perceptual inference and **(B)** active inference as suggested by the predictive coding model. The hierarchical structure of the prediction and prediction errors and their interaction can be seen in both inferences. Red, green, and blue arrows depict backward propagation of predictions, forward propagation of unexplained prediction errors, and interaction between predictions and prediction errors for explaining away remaining prediction errors, respectively. Light colors show that the corresponding signal is attenuated. P, predictions; PE, prediction errors.

Next, we focus on how modeling the world can result in the perception of different types of signals by the agent or movement of the agent in the environment (Figure 1). Our brain “must discover information about the likely causes of impinging signals without any form of direct access to their source” (Clark, 2013, p. 183); therefore, all inferences must be based on the changes in internal states, such as the state of light-sensitive receptors (Clark, 2013). Since not all data coming from the sensory organs can be analyzed all the time, the agent needs an efficient way to handle this monumental task. An economically efficient way of tracking the sensory input is first to predict the next state and then to encode what deviates from the predictions, or in other words, is surprising (Friston and Price, 2001; Friston, 2010; Friston, 2012). Indeed, the inception of predictive coding in engineering (Elias, 1955) was based upon the most efficient compression of sound files. In other words, one can also view predictive coding as finding efficient and compressed representations of (the causes of) sensory data (Schmidhuber, 2010), which speaks to the parsimonious way in which we encode our world.

Surprise, or prediction error, signals newsworthy events, which can be low- or high-level (Clark, 2013; Barron et al., 2020). For instance, the color of a black bag lying on a white table cannot be predicted from the surrounding color. Thus, there will be a low-level prediction error at the edge of the bag, where the color changes from white to black. Also, the agent might predict that this bag should be in the closet, and seeing it on the table may cause a high-level prediction error. In both cases, encoding prediction errors instead of raw information is more economically efficient (Clark, 2013).

Any agent needs to minimize the long-term average of surprise, which is best described as minimizing entropy (Clark, 2013; Friston et al., 2020); otherwise, the organism will succumb to the second law of thermodynamics (or its generalizations to open systems), meaning it cannot sustain its essential variables within physiological bounds. Based on the agent’s goal, the surprise can be resolved in two different ways. If the agent’s goal is to perceive external signals, prediction errors are more integral than predictions (Friston, 2010; Friston, 2012; Clark, 2013). In our table-and-bag example, sending the prediction error regarding the unpredicted black color on the white table upward in the system causes the predictions to be updated: there is a black bag on the white table. Notably, this process, called perceptual inference (Figure 1A), starts with predictions, and then, the violation of predictions indicates newsworthy information, initiating updating of the predictions (Friston and Price, 2001; Friston, 2010; Friston, 2012). In this scenario, predictions should have a lower weight than prediction errors; otherwise, similar to any other Bayesian inference, predictions will not be updated based on evidence. This increase in the relative weight of prediction errors to the weight of predictions can happen by increasing the “gain” of prediction errors in the system, e.g., by changing the focus of attention to the bag on the table (Auksztulewicz and Friston, 2015). This gain corresponds to the precision afforded prediction errors. In other words, the brain’s best guess about the reliability or confidence that can be associated with the information they convey. Physiologically, this can be understood as the postsynaptic gain or excitability (i.e., the rate constants) that govern neuronal dynamics (in the exchange between prediction errors and predictions encoded by various neuronal populations). Psychologically, an increase in precision is usually associated with selective attention, while a decrease in precision corresponds to sensory attenuation (Hohwy, 2012). Physiologically, the ability to predict the precision of precision-weighted prediction errors has been associated with mental action and the distinction between phenomenological transparency and opacity (Limanowski and Blankenburg, 2013; Limanowski, 2017). We will refer to these top-down predictions of precision in both physiological and psychological terms in what follows.

In contrast to perception, if the agent moves, predictions need to be enforced until the prediction errors are resolved (Adams et al., 2013; Brown et al., 2013), which is called active inference (Figure 1B). For instance, in our bag-and-table example, if the agent intends to grab the bag, it will produce predictions regarding the somatomotor (proprioceptive and somatosensory) signals coming from its hand. In the beginning, the predictions will not be aligned with afferent information (Adams et al., 2013): the hand should move toward the object, but it is static at first. However, instead of updating predictions based on prediction errors (i.e., perceptually inferring that its hand is static), predictions will be stubbornly forced until the ensuing prediction errors are resolved via reflex arcs (Yon et al., 2019). In other words, instead of proprioceptive prediction errors ascending the spinal cord and sensorimotor hierarchy to change predictions, they are used to drive neuromuscular junctions as part of classical motor reflex arcs (Brown et al., 2013). Therefore, descending predictions can be read as prior intentions that are realized in the periphery, provided ascending prediction errors are attenuated. This is usually associated with the phenomenon of sensory attenuation (Brown et al., 2013), which accompanies any self-generated act. In other words, to move is to ignore sensory evidence that one is not moving. Although this is a conceptually different way to eliminate surprise, it still follows the principles of the PCF: through the interaction between backward propagating predictions and forward propagating prediction errors, the long-term average surprise is minimized (Adams et al., 2013; Brown et al., 2013).

In the following, we will propose SATH as a framework that expands the PCF to account for the effects of direct verbal suggestions, including discussed hypnotic phenomena. We will further discuss how SATH can explain this broader range of hypnotic phenomena without internal contradictions.

## 4 Simulation-adaptation theory of hypnosis (SATH)

SATH is theoretically based on the PCF. Therefore, its fundamental assumption is that suggestion-induced responses are closely associated with top-down predictions and their interactions with somatosensory evidence. This emphasis on top-down cognitive processes is in line with the prevailing perspective in the literature (Terhune et al., 2017). Further, SATH claims that a cooperative and willing participant can employ three top-down processes for responding to direct verbal suggestions. Notably, the successful response to direct verbal suggestions refers to both objective and subjective aspects. The three postulated top-down processes are (I) *cognitive simulation* (for review, see Hesslow, 2002): simulating visual, auditory, or tactile stimuli can induce perceptual and neural processes similar to experiencing the corresponding stimulus in reality; (II) *neural adaptation* (for review see Lopresti-Goodman et al., 2013; Frank, 2016): top-down attenuation of sensory input can alter perception, causes among others, analgesia or agnosia; (III) *learning through simulation* (*cf.* Zahedi et al., 2020b): by mentally simulating an environment, novel strategies can be practiced, and consequently context-dependent trigger-response contingencies can be learned. These three top-down processes can be employed to different extents and in different combinations, depending on the individual capabilities, environmental cues, and other antecedents.

Before delving into the details of the theory, we need to address why, despite our focus on parsimoniousness, we proposed a tripartite theory. The rationale is twofold. First, previous factorial analyses of hypnotic suggestibility have shown that multiple groups of suggestions depend on correlated but distinguishable latent factors (Hilgard, 1965b; McConkey et al., 1980; Woody et al., 2005; Zahedi and Sommer, 2022). This fact is further pronounced when one considers that no single personality trait or cognitive process correlates more than moderately with suggestibility (Dienes et al., 2009; Lynn et al., 2019). Second, mounting evidence suggests there are multiple groups of highly hypnotizable participants who rely predominantly on different cognitive processes for responding to suggestions (Pekala et al., 1995; Barrett, 1996; Terhune et al., 2011; Terhune, 2015). Considering these results, any successful theory of suggestibility is required to reflect these heterogeneities by assuming more than one underlying cognitive process.

Next, we will address how SATH accounts for hypnotic phenomena in three areas. First, we suggest cognitive stimulation and top-down attenuation of sensory inputs as mechanisms underlying *motor responses* triggered by direct- and challenge-ideomotor suggestions and discuss alterations in the SoA during these movements. Second, we address suggestion-induced alterations in *perception* and the sense of conviction accompanying these changes. Third, we will explain how cognitive simulation can serve as a sophisticated mental simulator for training skills, accounting for the effects of task-relevant direct verbal suggestions on *executive functions* and *decision-making*. Finally, we will address hypnotic suggestibility and its correlates, such as social, psychological, and cognitive variables. Since the current article is not intended to be a review of theories of hypnosis (for narrative and systematic reviews, see Sheehan and Perry, 1976; Lynn and Rhue, 1991; Zahedi et al., 2021; Geagea et al., 2024), we only briefly discuss two hypnosis theories that are based on PCF at the end.

### 4.1 Motor suggestions

Motor suggestions are among the most common direct verbal suggestions, which is reflected in their prevalence in standardized hypnotizability scales, such as HGSHS-A (Shor and Orne, 1962) and SHSC-C (Weitzenhoffer and Hilgard, 1962). Although the performed actions are common everyday activities (e.g., levitating hands), two properties set them apart: (A) they are accompanied by resilient alterations in the SoA and SoR (Spanos and Barber, 1968; Kihlstrom, 2008), and (B) they are fluctuant, hesitant, and non-smooth (Martin and Pacherie, 2019). As explained above, motor suggestions can be divided into at least two categories: direct- and challenge-ideomotor suggestions; we will discuss these categories separately.

#### 4.1.1 Direct-ideomotor suggestions

Direct-ideomotor suggestions are responded to more often than any other type of direct verbal suggestions, as shown by item-response analyses (McConkey et al., 1980; Näring et al., 2004; Zahedi and Sommer, 2022). The term “ideomotor” refers to the ideomotor theory (for review, see Shin et al., 2010), which holds that thinking of the (perceptual) effects of a physical movement, which are retained and internalized through repetitions, will induce a tendency to produce that movement (Hommel et al., 2001). For instance, in the study of Elsner and Hommel (2001), participants repeatedly experienced a fixed co-occurrence between right and left button presses and low- and high-pitched tones, respectively, during a training phase. In the following test phase, low- and high-pitched tones preceded responses. The results indicated that the effects of a response (low and high tones) can promote the activation of the corresponding right and left button presses. Follow-up neuroimaging studies showed that response activations were correlated with the activation of premotor and somatosensory cortices (Melcher et al., 2008, 2013). Note that active inference formulation of motor control in the PCF is, effectively, a formalization of ideomotor theory. In other words, motor behavior is simply the realization of motor intentions, prior beliefs, or unattenuated predictions.

Can one propose that direct-ideomotor suggestions cause a motor movement because they force the participant to think about the perceptual effects of the movement? There are two issues here; first, although thinking about the perceptual effects of a movement induces a tendency to perform the movement, the tendency by itself rarely causes a full-fledged movement (Elsner and Hommel, 2001). This observation contrasts with what happens in response to direct-ideomotor suggestions, which, as discussed above, induce observable movements in most participants (Shor and Orne, 1963; Woody et al., 2005). Second, even in cases where thinking about the perceptual effects of a movement induces that movement, the movement is not accompanied by a reduced SoA, as is the case for direct-ideomotor suggestions (Blakemore et al., 2003). If anything, priming causes an increase in the SoA, meaning that participants become more prone to attribute others’ actions or accidental events to themselves (Aarts et al., 2009), which contrasts with a decrease in the SoA observed in participants responding to direct verbal suggestions (Lynn et al., 1990; Kirsch and Lynn, 1997; Blakemore et al., 2003).

To explain direct-ideomotor suggestions, we will first discuss the PCF’s account of altered states of consciousness, such as dreams and intentional imagery (Friston et al., 2020). By combining basic elements of the PCF, we will then propose a mechanistic account of direct-ideomotor suggestions.

**Figure 2:**
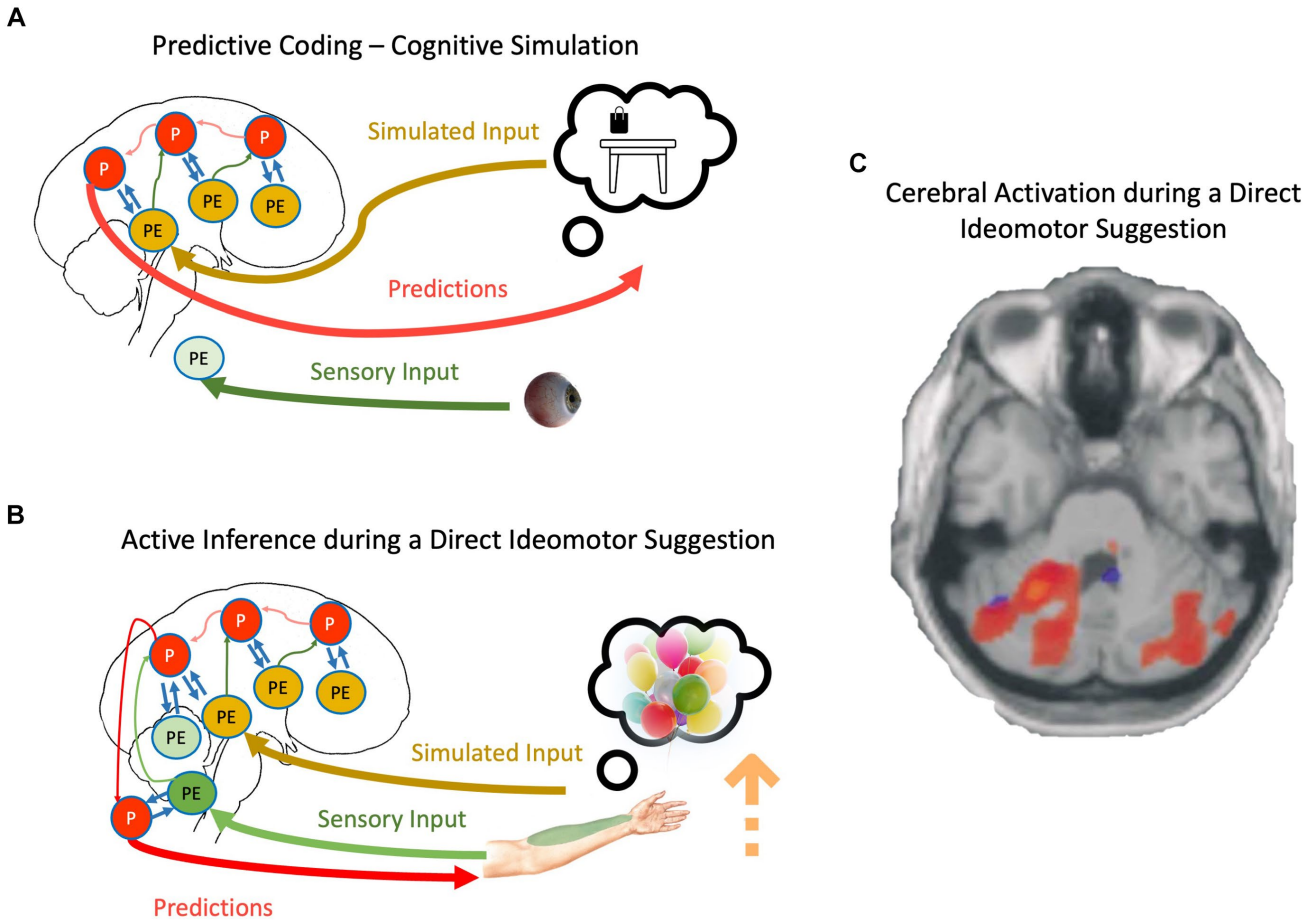
**(A)** The schematic representation of cognitive simulation using the principles of the PCF. **(B)** The schematic representation of direct-ideomotor suggestions. Red, green, yellow, and blue arrows depict backward propagation of predictions, forward propagation of unexplained prediction errors from sensory input, forward propagation of unexplained prediction errors from simulated reality, and interaction between predictions and prediction errors for explaining away remaining prediction errors, respectively. Light colors show that the corresponding signal is attenuated. P, predictions; PE, prediction errors. For a detailed explanation of processes, please look at the text. **(C)** Cerebellar activations in the Active Movement (blue) and Deluded Passive Movement (red) conditions. Activations in the cerebellum are more widespread in the Deluded Passive Movement condition compared with the Active Movement condition [adapted with permission from Blakemore et al., 2003].

In the PCF, not only are perception and imagery closely related (Kirchhoff, 2017), but also cognitive simulation (for review, see Farah, 1988; Hesslow, 2002; Figure 2A), which is a broader form of imagery, provides the basis of perceptual and active inference (Fletcher and Frith, 2009; Adams et al., 2013). Notably, cognitive simulation is broader than imagery as it is composed of proprioceptive, interoceptive, or extroceptive signals. Cognitive simulations of external and internal events are the output of the agent’s generative models and, therefore, closely tied to its predictions (Kirchhoff, 2017). Notably, intentional imagery is similar to other forms of altered states of consciousnesses, such as sleep and dreaming (Hobson and Friston, 2012; Friston et al., 2020), in the sense that in the absence of any sensory feedback, the agent is engaged in minimizing the complexity of the generative model via reducing the disparity between the posterior and prior beliefs (Friston et al., 2020). This process has a quintessential side-effect that we use in our mechanistic explanation of direct-ideomotor suggestions: Through these housekeeping-like activities, the precision of predictions will be increased as the redundancy in generative models is eliminated (Figure 2A).

Three questions need to be addressed: First, how is imagination maintained if the predictions are not aligned with the current sensory information? In other words, if imagery is not aligned with somatosensory input, it should cause sustained and uncorrectable prediction errors and interrupt imagery, which is not the case. As discussed before, the brain makes second-level predictions regarding the precision of its first-level predictions and prediction errors. During imagination, lower-level prediction errors are assigned a low gain (i.e., the agent does not attend to sensory information) since imagination is not expected to be aligned with sensory feedback. Therefore, during imagination, sensory information cannot perturb imagination (Jones and Wilkinson, 2020).

The second question that needs to be addressed is how the content of imagination is constrained. For instance, the content of imagination is coherent, and in most cases, it follows basic laws of physics (e.g., gravity). Similar to other altered states of consciousness, such as memory retrieval (Barron et al., 2020) or sleep (Hobson and Friston, 2012), during imagery, the hippocampus likely activates the neocortex and, subsequently, forms a stream of virtual information (Jones and Wilkinson, 2020). This offline stream of virtual information allows for unfolding comparisons between predictions and prediction errors and forms the basis of the coherence in imagery (Kirchhoff, 2017). Further, as the predictions come from heuristic models, they are aligned with previous events and, hence, follow the physical laws familiar to the agent (Jones and Wilkinson, 2020). However, imagery is *generative* by definition, and therefore, there are some deviations from the past. These deviations depend on the agent’s goals (e.g., imaging a planet without gravity requires deviation from heuristic models), context (e.g., when one expects to imagine bizarre geometrical shapes), and other antecedents (e.g., transitory states such as mood and hunger).

The third question is why we do not act out our imaginations. To address this question, we should consider the difference between imagery and perceptual or active inference. Perception and actions need to happen online, meaning that predictions evolve based on the ongoing stream of information. In contrast, during cognitive simulation, similar to memory retrieval or sleep, predictions are not compared to any sensory evidence; therefore, cognitive simulation happens offline (Hobson and Friston, 2012; Jones and Wilkinson, 2020). The offline property of intentional imagery, similar to dreaming (Hobson and Friston, 2012), ensures that the motor predictions are not backward-propagated to certain muscles. To understand this better, we will focus on dreaming; during the rapid eye movement (REM) part of sleep, where dreams occur most commonly, the anticipatory motor predictions are easily detectable (Cirelli and Tononi, 2008; Hobson, 2009). Accordingly, one should expect these anticipatory motor predictions (Hobson, 2009; Hobson and Friston, 2012) to provoke movement. However, motor inhibition during sleep prevents the agent from acting out its dreams (Hobson, 2009; Hobson and Friston, 2012). Therefore, the offline property of these altered states of consciousness is not due to the absence of motor predictions but due to active motor inhibition. Motor inhibition occurs by preventing motor predictions from reaching targeted muscles through top-down attenuation of prediction errors beyond thalamic nuclei.

Supporting this account, multiple studies show that imagining a stimulus not only activates similar brain areas but also causes the same responses as perceiving the corresponding stimulus in reality (for review, see Hesslow, 2002). For instance, imagining consuming a particular food, such as cheese, induces habituation (like its actual consumption) and, therefore, decreases the tendency of participants to consume similar items (Morewedge et al., 2010). Further, imagining performing an action will activate the same premotor and supplementary motor cortices as executing the action; the only difference is that the imaginary action does not activate the primary motor cortex, at least not as strongly as executing the action (for review, see Hesslow, 2002). Given that the primary motor cortex is involved in forming and backward propagating motor predictions (Adams et al., 2013), it is understandable that imagery of a movement does not result in the actual movement due to motor inhibition but recreates its sensory effects (Hesslow, 2002).

We can now adapt the mechanisms discussed regarding cognitive simulation and propose a mechanistic explanation of direct-ideomotor suggestions. During the *hand levitation suggestion* (Figure 2B), the participant is asked to imagine helium-filled balloons attached to his/her hand and then concentrate on somatosensory signals coming from the targeted hand, such as temperature and proprioceptive input (Hammond, 1990; Hammond, 1998). As the suggestion directly asks the participant to imagine the scenario, it is conceivable that he/she engages in cognitive simulation. This simulation should not necessarily be imagery but can be related to the retrieval of proprioceptive feedback during such a scenario (Hesslow, 2002; Shin et al., 2010). In any case, the precision of predictions will be increased due to the basic property of cognitive simulation, that is, aligning priors and posteriors and reducing redundancy in the generative models (Friston et al., 2020). However, unlike normal cognitive simulation, the participant expects to act out their imagination, and therefore, the cognitive simulation is not accompanied by top-down attenuation of motor predictions. Simultaneously, participants are repeatedly asked to attend to their somatosensory input from the targeted hand, which prevents sensory attenuation during the movement. Based on what we described regarding active inference, the participant should be unable to act out their motor predictions if somatosensory prediction errors are allowed to backpropagate beyond reflex arcs (Adams et al., 2013). The key is in unusually precise predictions resulting from cognitive simulations that can win against prediction errors even in the absence of sensory attenuation. The scenario is closely related to mechanisms underlying the delusion of alien control in schizophrenic patients: Unusually precise predictions, even while sensory attenuation is disrupted, result in movement accompanied by a disrupted SoA (Brown et al., 2013; Sterzer et al., 2018).

The account presented here is mechanistically similar to Edwards et al. (2012), who argue that unusually precise predictions due to increased attentional allocation, besides hierarchical dysregulation of sensory attenuation, cause the symptoms observed in hysteria. However, unlike hysteria where attentional processes are underlying the effects, we argue that cognitive simulations during suggestions and accompanying pruning-like activities will cause unusually precise predictions.

Can the SATH proposition account for the two properties of responses to ideomotor suggestions discussed earlier? First, why is the movement in response to direct-ideomotor suggestions hesitant and fluctuant (Kihlstrom, 2008; Martin and Pacherie, 2019)? As discussed, SATH assumes that motor predictions can win against unhindered prediction errors only if they become unusually precise through cognitive simulation. Aligning priors with posteriors through cognitive simulation increases the precision of predictions but is costly in terms of energy consumption, which is aversive (Hockey, 2011; Shenhav et al., 2017). Consequently, it is reasonable that the participant cannot engage in simulating the scenario far in the future, and at each point, they should focus only on the near future. This temporal restriction can create stepwise and hesitant movement observed in response to direct-ideomotor suggestions. Second, why are these movements accompanied by an altered SoA and SoR (Spanos and Barber, 1968; Frith et al., 2000; Kihlstrom, 2008; Martin and Pacherie, 2019)? SATH claims that the SoA is changed precisely because the somatosensory input is not attenuated. Since attenuation of somatosensory feedback is a vital element that the participant uses for inferring SoA (Brown et al., 2013; Sterzer et al., 2018), in the absence of sensory attenuation, the participant should have issues in attributing their actions to themselves. Further, based on SATH’s proposition, the agent is engaged in acting out their cognitive simulations. Although generative models constrain these simulations, they diverge from habitual physical laws such as gravity in the hand levitation example. Although aligned with generative models, these changes from habitual physical laws differentiate the situation from daily circumstances, creating the altered SoR accompanying responses to direct verbal suggestions (Bowers et al., 1988; Kihlstrom, 2008).

When discussing direct-ideomotor suggestions, we assumed positive expectations regarding executing the encountered direct verbal suggestion by the participant. This idea is supported by studies of Spanos et al. (1985) and Lynn et al. (1984), where two groups of highly hypnotizable participants were to resist hypnotic suggestions; one group was informed before hypnosis that good subjects could not resist suggestions, and the other group was informed to the contrary. Interestingly, the latter but not the former group could resist the suggestions. These findings indicate that having positive expectations regarding acting out the encountered suggestion is vital for freeing cognitive simulation from motor inhibition that usually accompanies it (Hobson and Friston, 2012; Friston et al., 2020).

Neuroimaging studies provide some preliminary evidence supporting SATH’s proposition regarding direct-ideomotor suggestions. For instance, a meta-analysis of neuroimaging studies investigating direct verbal suggestion (Landry et al., 2017) showed that one of the most reliable observations in the field is activation of the lingual gyrus while responding to hypnotic and posthypnotic suggestions. The lingual gyrus is a part of the visual system and is critically involved in imagery (Jung et al., 2016). Further, Blakemore et al. (2003) showed that activity in the contralateral cerebellum and bilateral parietal operculum areas increased when responding to direct-ideomotor suggestions compared to active and passive movement (Figure 2C). This result corroborates the account of SATH that prediction errors are not attenuated when responding to direct-ideomotor suggestions. In contrast, the unusually precise predictions due to the reduction in redundancy during cognitive simulation, despite precise prediction errors, cause the suggestion-induced movement.

#### 4.1.2 Challenge-ideomotor suggestions

As discussed above, challenge-ideomotor suggestions are the second form of direct verbal motor suggestions that aim to inhibit a motor response despite a secondary suggestion to neglect the primary suggestion. Challenge-ideomotor suggestions are more demanding than direct-ideomotor ones (McConkey et al., 1980; Näring et al., 2004; Zahedi and Sommer, 2022). As a canonical example, we will focus on the arm rigidity suggestion. In this suggestion (Shor and Orne, 1962; Shor and Orne, 1963), after asking the participant to stretch their arm in front of them and make a fist, the hypnotist will continue: “I want you to pay attention to this arm and imagine that it is becoming stiff… rigid like a bar of iron and how impossible it is to bend a bar of iron like your arm. Test how stiff and rigid it is. Now, try to bend it” (47[p. 9]). Notably, the main difference between direct- and challenge-ideomotor suggestions is that during direct-ideomotor ones, the participant is asked to focus on the part of the somatosensory feedback that is aligned with their cognitive simulation. For instance, during hand levitation, any alteration in the temperature of the hand or proprioceptive feedback regarding hand movements corroborates the feeling of lightness. In contrast, when the participant is asked to try to bend their arm during the arm rigidity suggestion, two sources of information clash: one from actual somatosensory feedback (i.e., the arm can be bent) and the other from virtual somatosensory feedback created by cognitive simulation (i.e., a bar of iron cannot be bent).

For understanding challenge-ideomotor suggestions, the concept of negative hysteresis (Frank, 2016) and how it relates to top-down processes is of great importance. In short, negative hysteresis refers to altered decision-making thresholds that are used for cognitive simulation compared to perception. A good example of negative hysteresis is provided by Lopresti-Goodman et al. (2013); they asked two groups of participants to judge whether they needed one or two hands to grasp wooden planks of different sizes. Participants in the control group actually grasped the planks, whereas the experimental participants merely saw the planks but were not allowed to touch them. Instead, participants in the experimental condition verbally reported whether they would need one or two hands. Importantly, planks were presented one by one in both ascending and descending orders. In the control group, the plank size, at which participants changed from one to two hands or vice versa, was slightly (but non-significantly) larger for ascending than descending presentation order; this numerically positive difference is called positive hysteresis. In contrast, in the experimental group without physical contact with the planks, the change point was considerably smaller in the ascending than in the descending order; this numerically negative difference is referred to as negative hysteresis. Frank (2016) explained this phenomenon in the framework of a Lotka–Volterra–Haken model for two neural populations representing the alternative responses in the task: (A1) a one-hand population and (A2) a two-hand population. In the control group, which actually executed the grasps and showed positive hysteresis, the outcome was modeled as follows:


(1) $$\frac{d}{dt}A_{1}=\alpha_{1}A_{1}-A_{1}^{d}-\beta \;A_{2}^{d-1}\;A_{1};\frac{d}{dt}A_{2}=\alpha_{2}A_{2}-A_{2}^{d}-\beta \;A_{1}^{d-1}\;A_{2}$$

where, $\alpha_{1}$ , $\alpha_{2}$ , and β represent synaptic weights of intra- and inter-population connections; $\alpha_{1}$ and $\alpha_{2}$ are exponential growth factors describing the increase or decay of the population variables in the linear format, and β designates the inhibitory interaction between the populations; d captures nonlinearities in the system. Roughly speaking, we can interpret the dynamics in Equation 1 in terms of precision-weighted prediction errors. For example, if we associate *A* with prediction errors, then the synaptic weights ( $\alpha_{1}$ , $\alpha_{2}$ , and β ) correspond to the precision of prediction errors that, as we will see below, change adaptively over time. Please see Bogacz (2017) for a technical discussion of synaptic weights and recurrent connectivity that predict the precision of local prediction errors.

To account for negative hysteresis, observed in the experimental group of Lopresti-Goodman et al. (2013), the activities of the neural populations must be adapted due to the prolonged neural activity (Lopresti-Goodman et al., 2013; Frank, 2016). Here, $\alpha_{1}$ and $\alpha_{2}$ vary slowly across each repetition of perception as follows:


(2) $$\{\begin{array}{c} \alpha_{1}=L_{1}\left(n\right)-Y\\ \alpha_{2}=L_{2}\left(n\right)+Y \end{array};i=1,2,and\forall T>1;L_{i}\left(n\right)=L_{i}\left(n-1\right)-\frac{1}{T}\left(L_{i}\left(n-1\right)-\left(L_{i,0}-S_{i}\right)\right)$$

where γ designates the variable of interest in relative format (e.g., relative plank size), $L_{1}$ and $L_{2}$ denote the dynamic rest levels of growth parameters $\alpha_{1}$ and $\alpha_{2}$ , respectively. Further, $L_{1,0}$ , $L_{2,0}$ , $s_{1}$ , and $s_{2}$ define the resting levels after adaptation is completed ( $L_{1}$ and $L_{2}$ ), as determined by theoretical considerations and experimental observations, respectively. Finally, T denotes the time scale of adaptation [for further mathematical details, see Lopresti-Goodman et al., 2013].

By combining Equations 1, 2, negative hysteresis can be explained in terms of the adaptation of neural activity in the targeted population due to prolonged activity. For the simulating condition compared to the physical perception condition, in ascending order, the one-hand population increasingly adapts across repetitions and is dominated by the two-hand population at a smaller plank size. Conversely, in descending order, the two-hand population adapts across repetitions and will be dominated by the one-hand population sooner in the simulation compared to the perception condition. This opposite shift in the change points results in negative hysteresis. Why is prolonged neural activity relevant only for the cognitive simulation condition? In the simulation condition, participants form mental representations of perceived objects, maintain them in their working memory, and examine (manipulate) them to judge how they should be grasped. In contrast, the controls respond directly to their perceptions; thus, perceived stimuli are not transmitted into working memory. Hence, the attenuation of the adapting neural population is conceived as a top-down process, as it is related to attention allocation rather than to a disturbance in bottom-up processes. The idea that top-down processes regulate perception and can directly affect perceptual pathways starting from thalamic activities is not restricted to negative hysteresis and has been corroborated by many studies (for review, see Saalmann and Kastner, 2009) and also in non-human subjects (Manita et al., 2015).

The focus of negative hysteresis on prolonged neural activity caused by top-down predictions is integral to the SATH proposition regarding the effects of challenge-ideomotor suggestions. As mentioned earlier, when encountering challenge-ideomotor suggestions, SATH assumes that the participant has two streams of somatosensory signals: one from actual somatosensory feedback (i.e., the arm can be bent) and the other from virtual somatosensory feedback created by cognitive simulation (i.e., a bar of iron cannot be bent). Based on SATH, the participant allocates heightened top-down attention to the actual somatosensory feedback. Normally, the heightened attention would increase the gain of prediction errors and make them more precise; however, if the allocated attention is high enough, the increased activity in the corresponding neural pathways needs to be adapted, similar to neural adaptation during negative hysteresis (Lopresti-Goodman et al., 2013; Frank, 2016). One needs to consider that neural adaptation in the study of Lopresti-Goodman et al. (2013) is limited to the cognitive simulation condition, as participants have to transfer the predictions generated during cognitive simulation to working memory, causing a prolonged activity in the corresponding neural pathways. SATH proposes that during challenge-ideomotor suggestions, unusually heightened attention to actual somatosensory feedback will cause the same neural adaptation. Consequently, if prediction errors from actual somatosensory feedback become imprecise due to neural adaptation caused by unusually amplified attention, virtual somatosensory feedback can become the relatively more precise input. In this scenario, the virtual somatosensory prediction errors will force the system to update predictions and result in perceptual inference (Brown et al., 2013): in the arm rigidity example, the arm feels like a bar of iron that cannot be bent.

Notably, the proposed mechanism has similarities to dissociative experiences. The unusually amplified attention observed in people with higher dissociative tendencies (DePrince and Freyd, 1999; de Ruiter et al., 2003; Brewin et al., 2013) creates a setting where actual somatosensory prediction errors can become imprecise enough that it does not result in perceptual inference. In that case, cognitive simulation can create a second stream of prediction errors. The virtual somatosensory feedback might be the basis of augmented reality when the second stream uses some elements of actual somatosensory feedback or virtual reality when prediction errors from simulated somatosensory input are used in isolation. In both cases, the perceptual inference should be due to prediction errors formed based on simulations rather than actual somatosensory information. It should be further noted that the amplified attention is not necessarily the result of the volitional allocation of attention but might be due to the inherent cognitive and neural characteristics of the participant, leading to higher attentional variability (Iacoviello et al., 2014) and diminished control over attentional processes (Aupperle et al., 2012), increasing attention biases toward actual somatosensory prediction errors (Fani et al., 2012).

Why are challenge-ideomotor suggestions more demanding and less responded to than direct-ideomotor ones (McConkey et al., 1980; Näring et al., 2004; Zahedi and Sommer, 2022)? Based on the proposition of SATH, in direct-ideomotor suggestions, somatosensory and simulated inputs are congruent, and therefore, neural adaptation, even if beneficial, is not necessary for responding to suggestions. On the contrary, in challenge-ideomotor suggestions, actual and simulated somatosensory inputs are incongruent, and therefore, the suggestion will be responded to, only if, neural adaptation due to amplified attention to actual input occurs, which makes them more demanding.

Although SATH propositions are mechanistic in explaining ideomotor suggestions, they can accommodate the usage of different strategies by different participants in responding to the same suggestion. Based on SATH, if suggestions are ambiguous about imagery, proprioceptive feedback, or settings that need to be simulated, participants are likely to come up with their own, which may have different consequences. For instance, Galea et al. (2010) investigated the physiological effects of the arm rigidity suggestion. To implement this suggestion, participants use divergent strategies, namely, (Schoenberger, 2000) some *simultaneously activated agonist and antagonist* muscles (biceps and triceps), (Alladin and Alibhai, 2007) some others *only activated the antagonist* (triceps) but inactivated the agonist, and some (Valentine et al., 2019) *did not activate any muscle* group. These results show that participants formulate different individual-specific predictions based on the same suggestion. Based on SATH’s proposition, if participants simulate the scenario where their arms are locked, they should activate the agonist muscle but simultaneously block the movement by activating the antagonist muscle. However, if they simulate the situation where their arms are temporarily paralyzed, they should only activate the antagonist muscle or not activate any muscle at all. Neuroimaging data partially supporting this account have been reported by Deeley et al. (2014). They reported that different (precise and elaborated) motor suggestions, focusing on a similar movement but consisting of different imaginations, caused different patterns of activation and functional brain connectivity. A part of these results show that neural correlates of suggestions depend on which cognitive simulations they are promoting, which is aligned with the proposition of SATH.

Further, one should note that SATH assumes a balance between cognitive simulation and neural adaptation and following sensory attenuation. That means, in line with previous results (Pekala et al., 1995; Barrett, 1996; Terhune et al., 2011; Terhune, 2015), we expect that some participants who are better at cognitive simulation require less sensory attenuation to achieve the dominance of suggested reality to actual reality, but some others who exhibit dissociative tendencies might rely more on sensory attenuation.

SATH can further provide a mechanistic explanation for neuroimaging studies focusing on challenge-ideomotor suggestions. For instance, based on SATH, one should expect that higher-order motor predictions would not be affected during direct verbal suggestions, but somatosensory prediction errors should be reduced due to neural adaptation, similar to negative hysteresis (Lopresti-Goodman et al., 2013; Frank, 2016). Accordingly, Ludwig et al. (2015) showed that hypnotic paralysis (similar to arm rigidity), in contrast to feigned paralysis, is not associated with decreased frontopolar cortex activity. However, in all conditions, the activity of premotor, motor, and somatosensory areas was reduced. Additionally, in an fMRI (Cojan et al., 2009) and follow-up EEG study (Cojan et al., 2013), it was shown that despite the preservation of preparatory motor predictions, the brain regions related to imagery, such as the precuneus and extrastriate visual areas, were more active during hypnotic paralysis compared to feigned paralysis. Further, frontal regions, especially the inferior frontal gyrus, were also more active during hypnotic paralysis than in control conditions, probably showing the amplified attentional allocation (Cojan et al., 2009, 2013).

One point needs to be discussed here. Zamansky and Clark (1986) showed that participants can respond to challenge-ideomotor suggestions even when they induce counter-imagination. For instance, while participants were responding to the arm rigidity suggestion, the hypnotist asked them to imagine being able to bend their arms. Interestingly, contradictory imaginations did not prevent medium and highly hypnotizable participants from following challenge-ideomotor suggestions. Does this finding contradict SATH’s proposition? SATH argues that the main driver of challenge-ideomotor suggestions is the neural adaptation caused by unusually strong attention to actual somatosensory input. Although a degree of cognitive simulation is required to produce a stream of virtual somatosensory feedback, this simulation is not necessarily imagery and can be related to the retrieval of proprioceptive effects of an event. Further, when considering the results of Zamansky and Clark (1986), it is evident that a contradictory suggestion was presented only if and immediately after hypnotized participants had successfully responded to the main challenge-ideomotor suggestion. In other words, only after successful neural adaptation during the first challenge suggestion was the second suggestion countering the primary suggestion presented. Therefore, these findings have little direct bearing against the propositions of SATH.

### 4.2 Changes in perception

Alterations in perception, induced by direct verbal suggestions, are commonly called “hallucinations” or “agnosia” to emphasize the strong conviction that participants develop about their responses (Kihlstrom, 2008). As discussed above, direct verbal suggestions can induce both positive and negative hallucinations, referring to adding elements to the perceptual reality or eliminating elements from it, respectively.

To explain perceptual suggestions via SATH, one does not need to assume any new mechanisms. However, when discussing positive or negative hallucinations, we are focusing on modalities where predictions are rarely, if ever, enforced in case of strong prediction errors (Sterzer et al., 2018; Yon et al., 2019). This feature contrasts with motor responses, where the active agent regularly uses sensory attenuation to eliminate prediction errors and enforce motor predictions (Adams et al., 2013; Brown et al., 2013; Clark, 2013). As a consequence, although the same two mechanisms employed for responding to ideomotor suggestions (i.e., sensory attenuation and cognitive simulation) are used for responding to perceptual suggestions, they constitute a separate category. To understand this point better, one can notice the difference between active and perceptual inference (Friston et al., 2020). Although active inference is closely related to internal states and the effects of changing internal states on external ones (i.e., the intrinsic information geometry), perceptual inference is focused on external states and, hence, the extrinsic information geometry (Friston et al., 2020). Therefore, this seemingly minor change in modality, i.e., from proprioceptive and motor predictions to exteroceptive ones, distinguishes perceptual suggestions from ideomotor ones.

**Figure 3:**
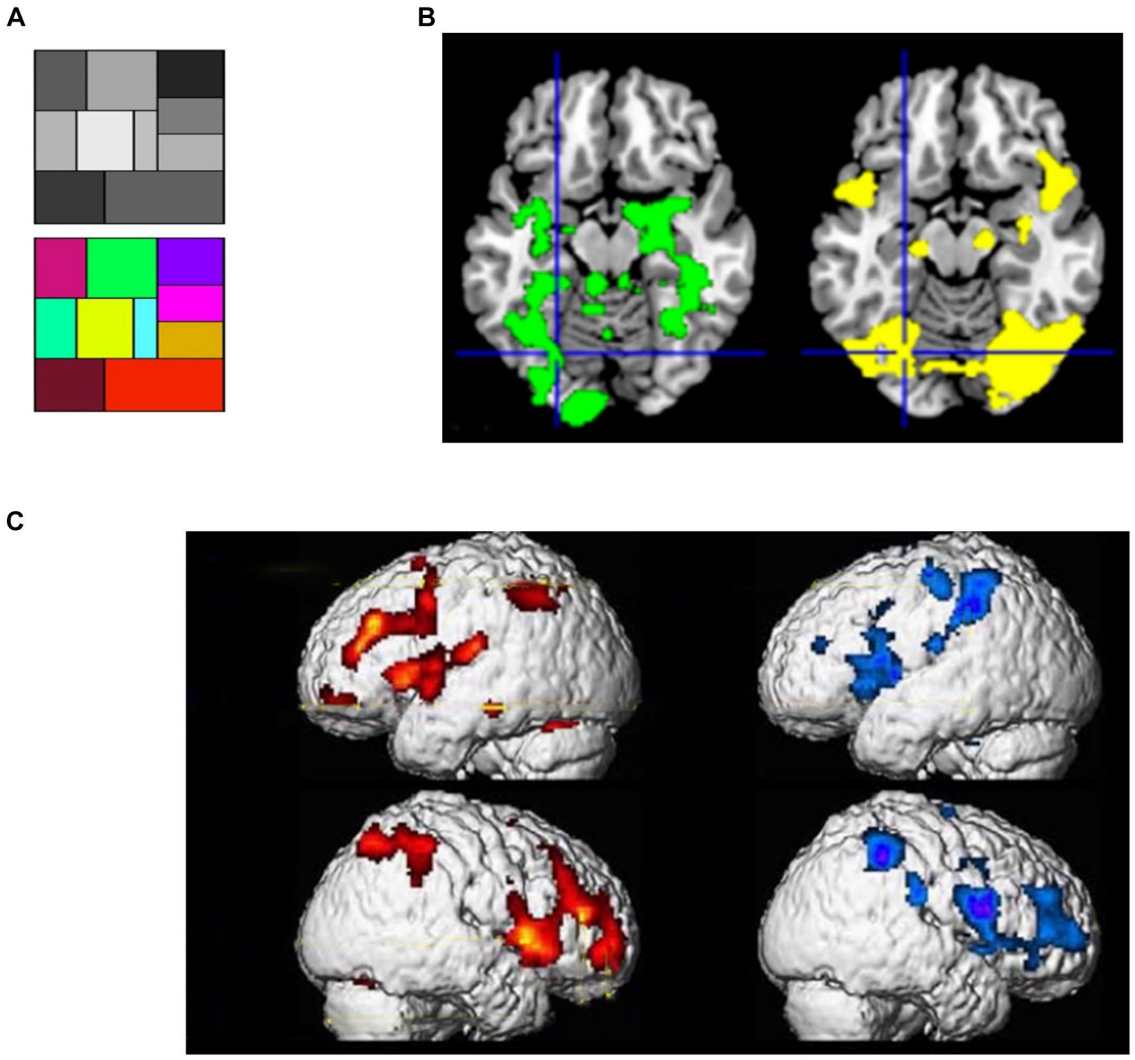
**(A)** Color and gray scales used in the study of McGeown et al. (2012). **(B)** (Left) the pattern of activation when viewing colors- compared to grayscale; (right) effects of a suggestion inducing positive color hallucination when looking at the grayscale in highly hypnotic suggestible participants; Crosshairs: the left fusiform region [adapted with permission from McGeown et al. (2012)]. **(C)** Brain activity of physically induced pain (left, red-yellow scale) and hypnotically induced pain (right, blue-purple scale) [adapted with permission from Derbyshire et al. (2004)].

If the proposition of SATH regarding the employment of cognitive simulation and sensory attenuation for responding to perceptual suggestions is correct, two sets of observations should be expected. First, if cognitive simulation creates predictions that are similar to perceptual inference for responding to perceptual suggestions, one should expect a comparable neural response to be caused by suggestions for positive hallucination and actual perception of stimuli. When different forms of positive hallucinations induced by suggestions are considered, such as auditory (Szechtman et al., 1998; Woody and Szechtman, 2000), visual (Mazzoni et al., 2009; McGeown et al., 2012), and tactile hallucinations (Derbyshire et al., 2004), they indeed cause the activation of the same brain regions as perceiving corresponding real stimuli. For instance, in an fMRI study, McGeown et al. (2012) first showed a grayscale and a color scale to their participants (Figure 3A); later, they showed the grayscale and suggested that participants mentally add color to the grayscale either inside or outside of hypnosis. Regardless of hypnosis, in highly hypnotizable participants, the suggestion induced the intended color hallucination, which was correlated with activity in color-sensitive brain areas (Figure 3B). Other fMRI recordings by Derbyshire et al. (2004) revealed that suggestion-induced pain and pain caused by physical stimuli activated similar brain areas, including the thalamus, ACC, insula, prefrontal, and parietal cortices (Figure 3C).

Two points should be discussed here. First, since SATH does not rely on the assumption of a specific state of consciousness for responding to hypnotic suggestions, it predicts the same effects for direct verbal suggestions regardless of hypnosis. Indeed, many studies showed that the effects of hallucination suggestions are similar to those delivered outside of hypnosis in terms of performance and brain activity (Mazzoni et al., 2009; McGeown et al., 2012).

The second point concerns the impact of expectations on determining the effects of cognitive simulation. Szechtman et al. (1998) tried to show that hypnotic suggestions and imagination affect hypnotized participants differently. They reported that in hypnotized participants, listening to a real sound and the hypnotic suggestion that a sound is present (their “hallucination condition”) lead to vivid impressions of hearing a sound. However, a hypnotic suggestion to “imagine” the sound did not cause the same report. Positron emission tomography (PET) imaging showed that the hallucination and real sound condition activated both the auditory temporal cortex and the ACC, whereas the imagination suggestion only activated the temporal cortex but not the ACC. A similar observation has been made in the study of Derbyshire et al. (2004), which used the method of Szechtman et al. (1998) and compared the effects of physically painful stimuli, the imagination of pain, and hypnotic suggestion-induced pain. Do these studies show that cognitive simulation cannot account for the effects of direct verbal suggestions? As discussed above, SATH’s propositions rely on positive and unhindered expectations of the efficacy of the suggestions. In the study of Szechtman et al. (1998), all three conditions took place inside of hypnosis and, hence, were induced by hypnotic suggestions. Therefore, differences between these conditions cannot be used to address the differences between pure imagination and hypnotic suggestions. In this special study, by contrasting different conditions, participants might have developed diverging expectations regarding the imagination condition versus hallucination or listening conditions. Since negative expectations can be particularly detrimental (Jones and Spanos, 1982; Green and Lynn, 2011), these results will not have direct bearings regarding SATH’s hypotheses.

The second set of expected observations based on SATH’s propositions are related to sensory attenuation of actual somatosensory input. Specifically, SATH predicts the sensory attenuation of actual somatosensory input happens when it contradicts hallucination suggestions. This proposition may be better understood when considering negative hallucination suggestions since they are focused on downregulating an actual sensory input. For instance, in the “three boxes” suggestion in the Sandford hypnotic susceptibility scale, three boxes are placed in front of participants, but the hypnotist informs them that there are only two (Weitzenhoffer and Hilgard, 1962). Here, we specifically focus on a common type of negative hallucination suggestions, namely pain-reducing suggestions, as they are successful in a majority of participants, and there is ample evidence about their neural underpinnings (for review, see Thompson et al., 2019).

There are numerous pain-reducing suggestions (for review, see Hammond, 1998); most of them ask participants to specifically attend to pain or form a mental representation of pain-eliciting stimuli and to manipulate this mental representation. For instance, participants might be asked to describe pain elicited by noxious stimuli in terms of a physical object (e.g., a balloon or a brick) and interact with this object (e.g., crunching it or reducing its size). Therefore, in contrast to normal conditions, where participants directly react to stimuli, these suggestions ask them to form a mental representation of noxious stimuli. The disparity between these two conditions is similar to the difference between cognitively simulating planks versus directly interacting with planks, discussed regarding the study of Lopresti-Goodman et al. (2013).

Let us assume that there are two neural populations with precisions $\alpha_{1}$ and $\alpha_{2},$ encoding prediction errors and predictions representing a stimulus, such as an ice cube on the skin, as harmless versus painfully cold. In normal situations with direct reactions to pain-evoking stimuli, Equation 1 only explains positive hysteresis. When a stimulus is previously judged as painful, the stimulus will be judged as still painful, with a slightly lower activation than for an isolated (novel) stimulus. This can be considered as becoming more sensitive to pain. Conversely, when reacting to the same stimulus after a pain-reducing hypnotic suggestion, two scenarios might happen. Either participants focus excessively on painful stimuli, or they form a mental representation of the stimulus and work on it (e.g., by judging its severity or trying to describe and manipulate it). Both scenarios cause an increased and prolonged neural activity in the pain perception population. Therefore, the growth parameter of the pain perception population, $\alpha_{2}$ , will be downregulated, as described in Equation 2. Consequently, the activation of the population based on which participants judge stimuli as painful will be decreased (negative hysteresis). This decrease in the activity of the responsible neural population results in the perception of the same stimulus as harmless. In other words, suggestions reduce pain by establishing a mental representation of pain or focusing on the painful stimuli, causing a prolonged neural activity that results in sensory attenuation. This mechanism is not restricted to pain-provoking stimuli. For instance, the perception of tactile, non-pain-provoking stimuli can also be affected by sensory downregulation via top-down processes. The only requisite is that participants either amplify their attention toward it or form a mental representation of the stimulus and engage in manipulating this representation rather than directly responding to sensory input (Vanhaudenhuyse et al., 2009).

**Figure 4:**
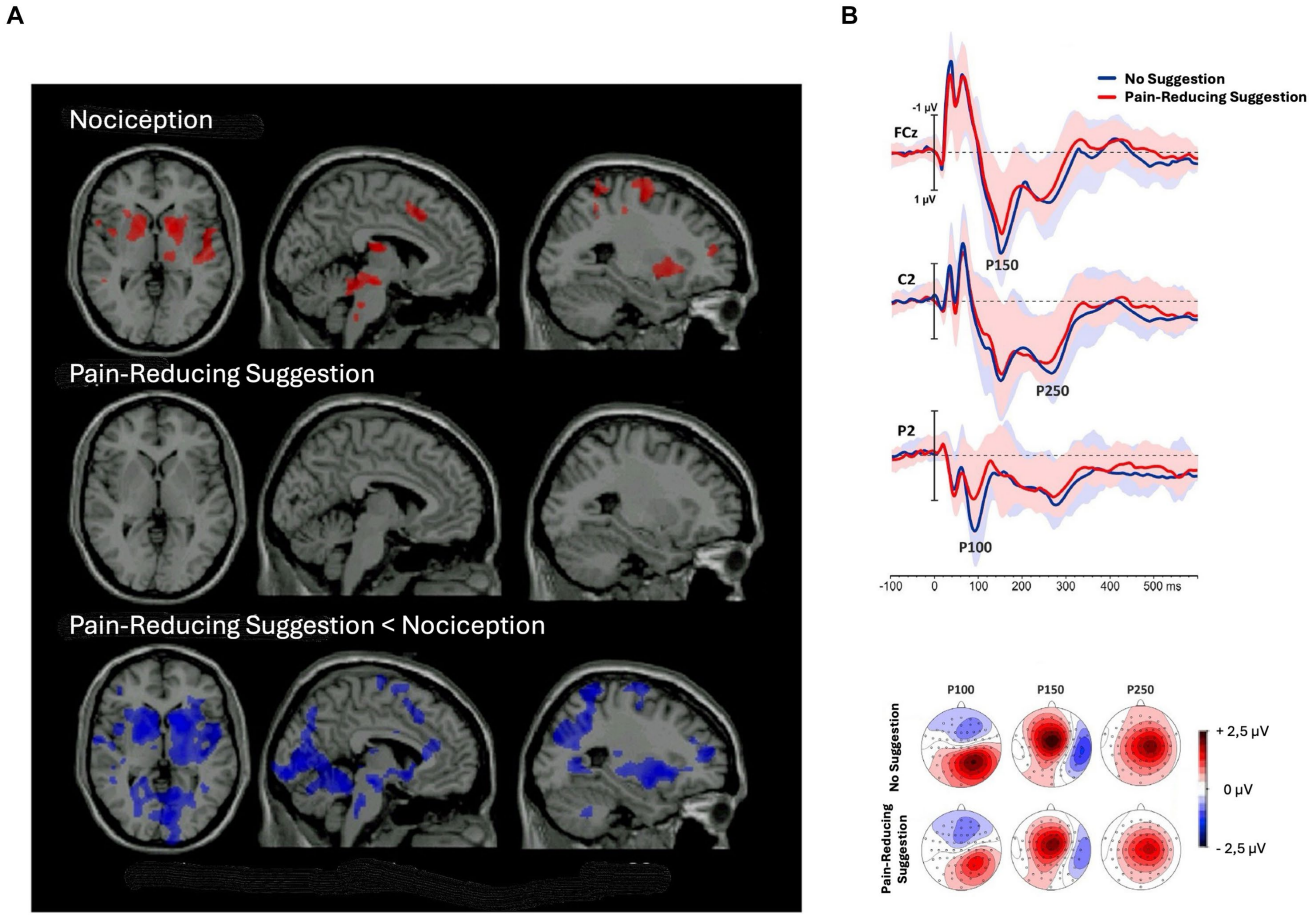
Decrease in brain activity after pain-reducing hypnotic suggestions. **(A)** Brain regions showing significant (*p* < 0.05) activation during noxious stimulation (upper row) without hypnosis, (middle row) under the influence of pain-reducing hypnotic suggestions, and (lower row) the hypnotic condition minus the no-hypnosis condition [adapted with permission from Vanhaudenhuyse et al. (2009)]. **(B)** (Top) grand-average waveforms of sensory-evoked potentials (SEPs) without hypnosis and during hypnosis; shaded areas represent standard deviations; (bottom) topographic maps of the P100, P150, and P250 components in the two conditions [adapted with permission from Perri et al. (2019)].

The adaptation account of negative hallucinations predicts that brain areas, being activated in response to noxious stimuli, will be less activated after receiving pain-reduction suggestions in comparison to normal conditions.^2^ This prediction is supported by both fMRI and ERP studies. For instance, in an fMRI study, Vanhaudenhuyse et al. (2009) found that all brain regions activated by pain perception, that is, brainstem, right thalamus, bilateral striatum, right primary somatosensory, bilateral insula, anterior cingulate cortex, right middle frontal gyrus, and right premotor cortex, showed less activation following pain-reducing hypnotic suggestions in comparison to a condition without hypnosis (Figure 4A). In an ERP study, Perri et al. (2019) found hypnotic suggestions to reduce ERP components correlated with pain perception, such as N20, P100, P150, and P250 (Figure 4B). Therefore, the top-down sensory attenuation of the neural population responsible labeling stimuli as painful may explain pain reduction due to hypnotic suggestions. Reasonably, sensory adaptation processes may also explain other negative hallucinations.

In generalizing SATH from pain to other perceptual suggestions, one should consider that sensory attenuation due to neural adaptation does not depend on the actual sensory information and external information geometry. In the end, the agent has only access to the output of its sensors rather than the actual world (Friston et al., 2020). For instance, similar to a person with out-of-body experience (Blanke and Metzinger, 2009), a person with extreme dissociative tendencies may preemptively cause sensory attenuation due to neural adaptation in the sensors, primary, and associated neural pathways, which might then lead to hallucinations in response to difficult suggestions such as the three-box suggestion discussed earlier.

#### 4.2.1 Sense of conviction

Why do participants develop a sense of conviction only in response to suggestions but not during normal imagination? Ganis and Schendan (2008) compared the ERP effects of imagining versus perceiving stimuli and showed that imaginations induced the same perceptual processes as external stimuli. The authors suggested that mental imagery causes mental representations of imagined stimuli to be formed and maintained in working memory. This procedure contrasted with the perception condition, in which perceptual representations are rapidly decayed. Ganis and Schendan (2008) concluded that due to the difference in the persistence of (mental vs. perceptual) representations, participants usually do not confuse mental imagery with the perception of external stimuli.

Let us consider the effects of positive hallucination suggestions first. During positive hallucinations, participants are asked to form a stream of mental images (e.g., a developing story rather than a single image). Therefore, participants do not form a single mental representation but a stream of representations, subjected to the same normal decay as external stimuli due to limited working memory capacity (Diamond, 2013). In other words, the unfolding cognitive simulation cannot be maintained in working memory and will be discarded after a certain time. Therefore, these representations can easily be confused with perceived real-life events due to this rapid decay. The distinction between normal daydreaming and the suggested processes in cognitive simulation lies in unusually precise predictions originating from pruning-like processes during cognitive simulation, which is unnecessary and unlikely to form during common daydreaming. Notably, pruning-like activities refer to aligning priors and posteriors and reducing redundancy in the generative models during cognitive simulation, which was discussed as an inherent characteristic of the process. Further, when responding to suggestions, cognitive simulations will be the source of predictions that are compared with virtual somatosensory prediction errors due to sensory attenuation following neural adaptation. Therefore, these processes will make these predictions unusually resource-consuming, leading to much faster decay than for the contents of common daydreaming.

In contrast to positive hallucination suggestions, during negative hallucinations, an external stimulus is transformed into a mental representation and manipulated repeatedly, causing an enduring representation with an accompanying neural activation that can be subjected to top-down attentional effects rather than normal sensory decay or attenuation. This neural adaptation causes an actual somatosensory input to be judged as imaginary. Therefore, when participants respond successfully to perception-related suggestions, the subjective sense of conviction about the mental imagery seems to be a byproduct of the underlying cognitive processes.

### 4.3 Changes in cognition

#### 4.3.1 Learning through simulation

A commonly administrated type of direct verbal suggestions is related to the suppression of habitual responses or to learning new stimulus–response contingencies. Here, we will first discuss how SATH accounts for the effects of task-relevant suggestions on performance in cognitive tasks, followed by the effects of neutral hypnosis (i.e., hypnosis without task-relevant suggestion). Briefly, SATH claims that the enhancing effects of suggestions on performing cognitive tasks can be attributed to improved learning of new stimulus–response contingencies and, consequently, more efficient implementation of cognitive control processes.

Many studies using posthypnotic suggestions to manipulate cognitive processes have focused on the inhibition function as required, for example, in the Stroop (Raz et al., 2006; Zahedi et al., 2019), Erikson (Iani et al., 2006), Simon (Iani et al., 2009), and Go-NoGo task (Zahedi et al., 2020a). In these tasks, performance enhancements might be attributed to both bottom-up or top-down processes (*cf.* Zahedi et al., 2020b). For instance, in the Stroop task, color words are presented in different ink colors. Participants are required to respond to the ink colors while ignoring words’ meanings. Here, a habitual response, that is, reading the word, has to be suppressed in order to avoid conflicts with naming the ink color. Consequently, Stroop task performance can be improved via (Schoenberger, 2000) alterations in bottom-up processes, such as blocking interfering semantic input to prevent any interference. Damage to the occipitotemporal region of the left hemisphere through which visual word forms are attained can cause a form of dyslexia characterized by letter-by-letter reading (Warrington and Shallice, 1980). In the same manner, if posthypnotic suggestions in the Stroop task can affect bottom-up processes, for instance, by decoupling or impairing the word-form system, task performance will be enhanced without employing cognitive control. Alternatively, (Alladin and Alibhai, 2007) participants may deploy additional cognitive control to detect and suppress interfering information more efficiently, which, in turn, facilitates conflict resolution. This second scenario, however, relies on implementing top-down processes.

Recent findings show that posthypnotic suggestions can also enhance performance in working memory updating tasks (Lindelov et al., 2017; Zahedi et al., 2020b), where changes in bottom-up processes cannot contribute to task performance (*cf.* Zahedi et al., 2020b). Therefore, the effects of hypnotic and posthypnotic suggestions may be specifically related to alterations in top-down processes (Terhune et al., 2017). However, which specific top-down processes can be affected by posthypnotic suggestions is a more contentious issue.

Usually, hypnotic and posthypnotic suggestions, which are used to improve performance in cognitive tasks, are merely elaborated rephrasings of task instructions. Thus, the effects of these suggestions cannot be attributed to implementing a different strategy (Zahedi et al., 2020b). However, when one considers cognitive tasks in general, they engage participants in novel situations requiring the development of new responses. Also, in many of these studies, hypnotized participants are asked to imagine the targeted task and implement suggestions in their imagination (Zahedi et al., 2017, 2020b). In these scenarios, cognitive simulation might provide a setting where multilevel associations can be mentally practiced. SATH makes an implicit assumption: cognitive control is related to multi-level learning (Egner, 2014), which involves associative learning related to both trial-by-trial and more abstract trial features (Abrahamse et al., 2016). Hence, cognitive learning can be understood in terms of creating associations between stimuli and responses that are context-dependent and modulated by rewards and punishments (Abrahamse et al., 2016). Further, environmental cues will inform the necessary rate of updating the models based on higher-order characteristics such as environmental stability (Trempler et al., 2017; Simoens et al., 2024). Noticeably, it has been shown that independent of hypnotic suggestions, the application of mental practice can enhance physical or cognitive skill-learning procedures (Frank et al., 2015; Stefanidis et al., 2017). Further, refuting the claim that only hypnotic and posthypnotic suggestions can affect performance, it has been shown that task-relevant suggestions can enhance cognitive performance also outside of hypnosis (Parris and Dienes, 2013; Palfi et al., 2020).

How does learning boost performance in inhibition and updating tasks? In inhibition tasks, a second well-learned stimulus–response association, which can compete with the automatic but inappropriate response, makes participants capable of exerting inhibition more efficiently (Dulaney and Rogers, 1994; Protopapas et al., 2014). For example, Stroop effects are resilient to practice but not immune and can be significantly reduced by extensive practice in participants of almost every age (Dulaney and Rogers, 1994; Protopapas et al., 2014). Learning, however, can happen on multiple levels (Egner, 2014), which will become clearer if one considers changes in conflict adaptability (Egner, 2014; Yang et al., 2022) and proportion congruent effects (Schmidt and Besner, 2008). Conflict adaptability refers to the observation that congruency effects will be reduced after an incongruent compared to congruent trial in the Stroop (Egner, 2014; Yang et al., 2022) or similar cognitive tasks (Sturmer et al., 2002; Sturmer and Leuthold, 2003). Notably, these modulations depend on trial-dependent feedback in the Stroop task (Yang et al., 2022). The proportion of congruent effect refers to the reduction of congruency effects when the proportion of incongruent to congruent trials is higher in different blocks (Schmidt and Besner, 2008; Mayr and Awh, 2009). These observations indicate how both lower- and higher-order associations can independently (Mayr and Awh, 2009) modulate cognitive control (Egner, 2014). In the same manner, mental practice during suggestions can affect these multi-level associations, which ultimately causes a reduction in congruency effects and, therefore, enhancements in task performance.

Additionally, it has been shown that extensive training can enhance performance in updating tasks but will not actually increase working memory capacity (Diamond and Ling, 2016). Instead, a well-learned response empowers participants to utilize their cognitive control processes more efficiently. To summarize, it has not only been shown that practice can enhance performance in inhibition and updating tasks but also that the mechanisms underlying these enhancements are the same as those mechanisms that SATH proposes for explaining the effects of task-relevant direct verbal suggestions (Zahedi et al., 2020b).

Two aspects of the effects of direct verbal suggestions need further consideration. First, direct verbal suggestions can be turned on and off by presenting a cue that had been mentioned in the suggestions (a process called anchoring) (Raz et al., 2003; Iani et al., 2006; Zahedi et al., 2020b). If learning and habitualization of a new stimulus–response association results in observed behavioral enhancements, one might expect they will be present even after deactivating the suggestion. It has been repeatedly shown that learning can be context-dependent, especially if learned responses are not extensively practiced across different settings. For instance, Abrahamse and Verwey (2008) have shown that changing the context causes participants to inhibit learned responses. In addition, Ruitenberg et al. (2012) showed that changing contextual cues can be detrimental to learned responses, especially if the duration of practice is limited. The same may be true for the effects of direct verbal suggestions. Especially if one considers that these suggestions do not cause an automatic response to be formed (Tobis and Kihlstrom, 2010), contextual dependencies can explain why the effects of direct verbal suggestions vanish when they are deactivated.

Second, what are the benefits of direct verbal suggestions if their effects can be understood in terms of practice? Practice-related enhancements in cognitive performance are often achieved through very extensive training sessions and confined to the trained cognitive skill (Diamond and Ling, 2016; Melby-Lervag et al., 2016). This contrasts suggestions, which can affect performance after a relatively short mental practice (Zahedi et al., 2020b) and target higher-order abstractions rather than trial-by-trial associations (Lindelov et al., 2017). Therefore, as discussed in several studies, suggestions can be used to improve the efficacy and efficiency of cognitive training in both normal participants (Zahedi et al., 2020b) and brain-damaged patients (Lindelov et al., 2017).

Notably, to explain the temporal properties of suggestion effects, SATH relies on the fundamental properties of cognitive simulation: while predictions are isolated from the somatosensory prediction errors (Kirchhoff, 2017; Jones and Wilkinson, 2020), predictions are aligned with simulated prediction errors, and thus, redundancy in the existing generative models will be reduced (Hobson and Friston, 2012; Friston et al., 2020). This process facilitates the formation of more precise predictions in shorter time periods and with less repetition. What happens during simulation is similar to the transference of content from short-term to long-term memory during sleep (Hobson and Friston, 2012; Friston et al., 2020): while the agent is isolated from the environment and therefore, no new memory is created, weaker connections are eliminated in favor of strengthening more prominent ones, (Barron et al., 2020; Jones and Wilkinson, 2020). Notably, the isolation from actual somatosensory input reduces the necessity for cognitive stability, i.e., protecting the models from being updated based on random noise, and thus, the agent can be cognitively more flexible and update the models more readily when faced with substantial evidence (Trempler et al., 2017; Dreisbach and Fröber, 2018). However, it should be noted that different participants might target differential contingencies via cognitive simulation. For instance, in the case of word-blindness suggestions in the Stroop task, some participants might modulate lower-level, trial-by-trial contingencies, which would affect performance similar to conflict adaptation (Egner, 2014; Yang et al., 2022) both in terms of magnitude and quality. Yet, other participants might modulate higher-order, context-relevant contingencies that have similar effects as the proportion of congruency (Schmidt and Besner, 2008).

**Figure 5:**
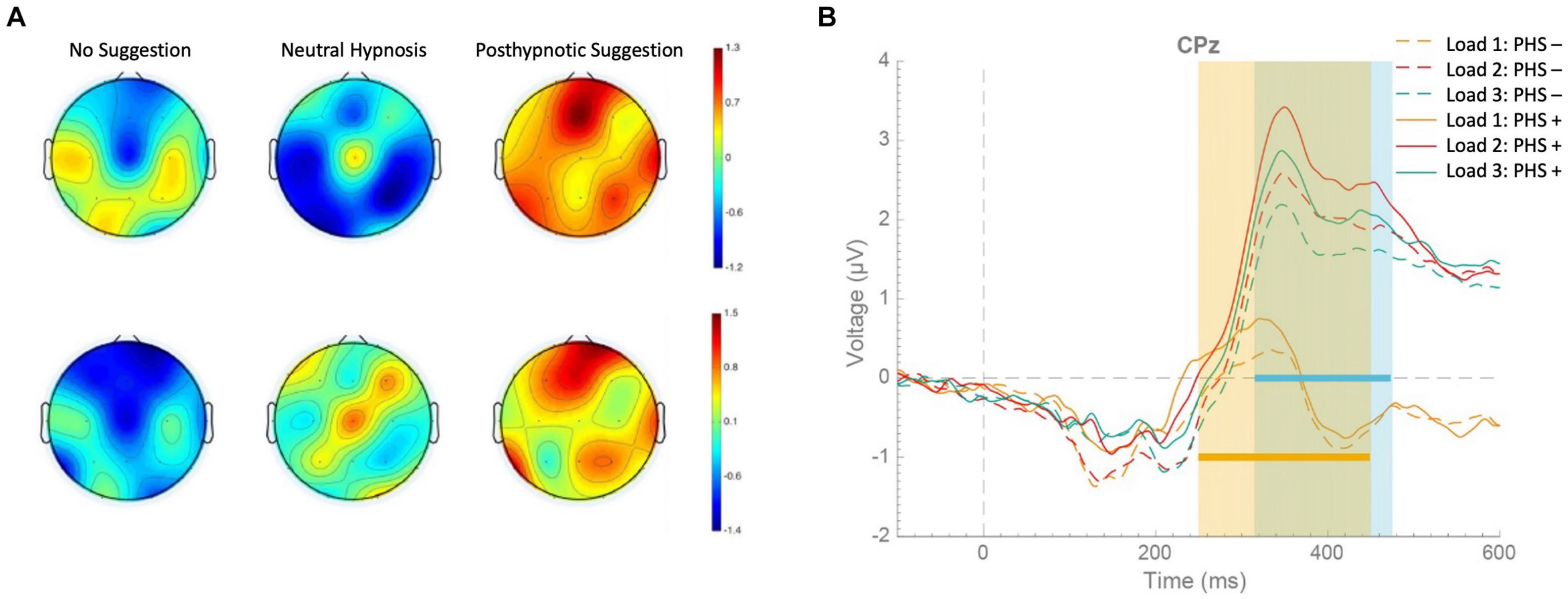
**(A)** (Top) Theta and (bottom) beta activation during the completion of the Stroop task [adapted with permission from Zahedi et al. (2017)]. **(B)** Increased P3 amplitude when posthypnotic suggestions are activated compared to deactivated during the completion of the tone-tracking task (Miyake et al., 2000), measuring updating in working memory; PHS-, posthypnotic suggestions are deactivated; PHS+, posthypnotic suggestions are activated [adapted with permission from Zahedi et al. (2020a)].

Suppose the effects of direct verbal suggestions are related to mental practice. In that case, performance enhancements in updating and inhibition tasks should be related to enhanced utilization of proactive control and decreased utilization of reactive control. Proactive control is a form of control recruited in advance of a situation where executive control might be necessary without consideration of its actual necessity. In contrast, reactive control is employed when the need for cognitive control, such as conflict resolution, has been detected (Braver, 2012). Several lines of results corroborate this hypothesis. First, Zahedi et al. (2017) observed that during task completion under the influence of suggestions, frontal theta and beta activity were increased (Figure 5A). This result possibly indicates increased utilization of executive functions (Reinhart and Nguyen, 2019) and reduction of prediction errors or the precision-weighting afforded to these predictions errors (Palmer et al., 2019) when suggestions are activated. Second, previous studies (Zahedi et al., 2019, 2020a,b) repeatedly showed that when direct verbal suggestions are activated, P3 amplitude is increased (Figure 5B). The increased P3 possibly highlights the incorporation of top-down processes and attentional resources (Polich, 2007; Fonken et al., 2020).

Finally, if the proactive hypothesis is correct, then when direct verbal suggestions are active, task load effects should decrease in both inhibition and updating tasks. Corroborating this hypothesis, Raz et al. (2005) observed that in inhibition tasks under the effects of direct verbal suggestions, conflict resolution improves, resulting in decreased brain activity in regions related to conflict detection, such as the ACC. Furthermore, under the influence of suggestions, the N400 amplitude was decreased (Zahedi et al., 2019), which shows a reduction in semantic activation caused by automatic word reading in the Stroop and similar tasks. Further, decreased activity in brain regions related to semantic activation, such as the fusiform gyrus, superior and middle temporal gyri, pre- and postcentral gyri, and supplementary motor area (Ulrich et al., 2015), corroborates the reduction in task load. Also, in updating tasks, the task load on working memory buffers decreased due to enhancements caused by direct verbal suggestions (Zahedi et al., 2020b).

Notably, neutral hypnosis has no reliable effect on performance in cognitive tasks (Egner et al., 2005; Zahedi et al., 2017). According to SATH, it is conceivable that only task-relevant suggestions, which can provide a ground for mental practice, may affect performance, and task-irrelevant suggestions, such as relaxation-inducing ones, presented during neutral hypnosis, will not affect performance in any systematic way.

#### 4.3.2 Changes in decision-making

As mentioned above, direct verbal suggestions can be used to affect participants’ decisions. One interesting application of suggestions is related to shifting participants’ food choices toward more healthy food items (Ludwig et al., 2014; Zahedi et al., 2020a, 2023). The suggestion that is used for this matter commonly asks participants to imagine a specific type of food and, by attending to its physical properties, imagine how delicious it is (Zahedi et al., 2020a).

Here, the account of SATH regarding learning through simulation can be expanded to explain the effects of direct verbal suggestions on decision-making. If, in response to other cognitive suggestions, participants need to create novel stimulus–response contingencies, in response to decision-making suggestions, they need to form stimulus-outcome associations (Colwill, 1993; Colwill and Delamater, 1995). In a sense, SATH proposes that the effects of direct verbal suggestions are similar to evaluative conditioning (Hofmann et al., 2010; Hutter and Rothermund, 2020), where emotionally neutral stimuli will become emotional through associations with unconditioned emotional stimuli. If one focuses on the food suggestions mentioned above, the only difference between standard evaluative conditioning and direct verbal suggestions is that in response to suggestions, participants need to simulate unconditioned and conditioned stimuli mentally. Further, as the simulation can be focused on abstract and semantic entities, the unconditioned stimuli will be broader compared to what one can use during standard evaluative conditioning. For instance, participants might directly associate a food category with deliciousness, which is a semantic entity rather than a standard unconditioned stimulus. Other than that, in both cases, emotionally neutral stimuli become conditioned through associations with unconditional stimuli.

Accordingly, previous studies show that direct verbal suggestions affect participants’ preferences for the targeted food items, which can explain changes in their choices (Zahedi et al., 2023). Further, the changes in preferences are related to the increased P1 amplitude (Zahedi et al., 2020a), which is an early ERP component that has been shown to be associated with preferences and reward saliency (Hickey et al., 2010; Donohue et al., 2016).

#### 4.3.3 Sense of conviction

As discussed before, the SoA has two subcomponents: involuntariness and effortlessness (Polito et al., 2013). Considering alterations in the SoA under the influence of direct verbal suggestions targeting performance in cognitive tasks, effortlessness is more relevant than the experience of involuntariness. In other words, since participation in a cognitive task requires goal-directed actions, participants cannot sense involuntariness, even if one is already well-equipped with appropriate responses. This can be translated into the feeling that suggestions cause better performance with the same effort as before rather than conducting an action without attributing it to the direct exertion of volition. As suggested by SATH, under the influence of direct verbal suggestions, participants may perform a cognitive task with less reactive cognitive control and more proactively and consequently more efficiently (Zahedi et al., 2019, 2020b; Parris et al., 2021), which would feel comparatively effortless.

### 4.4 Hypnotizability and its determinants

Participants are different in their responsiveness to hypnotic and posthypnotic suggestions. In other words, some are more hypnotizable than others (Shor and Orne, 1963; McConkey et al., 1980; Bongartz, 1985; Woody et al., 2005). Hypnotizability can be defined as what is measured by standardized scales of hypnotic susceptibility (for review, see Woody and Barnier, 2008). Another way to think about hypnotizability has been offered by Kirsch (1997), who distinguished between (I) suggestibility, (II) hypnotic suggestibility, and (III) hypnotizability. (I) Suggestibility is defined as the capability to respond to direct verbal suggestions outside of hypnosis. (II) Hypnotic suggestibility, on the other hand, is the ability to respond to direct verbal suggestions after hypnotic induction. Finally, (III) hypnotizability is the increase in suggestibility due to the induction of hypnosis. According to Kirsch (1997), common hypnotic susceptibility scales measure hypnotic suggestibility rather than hypnotizability *per se*. However, since there are strong correlations between general suggestibility and hypnotic suggestibility (r = 0.67 for behavioral scores; r = 0.82 for subjective scores; Braffman and Kirsch, 1999), measuring hypnotizability as defined by Kirsch (1997) is challenging.

SATH embraces the discussion of Kirsch (1997) and, hence, it distinguishes between general *suggestibility*, that is, the capability of a person to respond to suggestions regardless of hypnosis, and *hypnotizability*, that is, the increase in suggestibility due to the reception of hypnotic induction. Accordingly, the top-down mechanisms proposed by SATH are related to general suggestibility and not to hypnotizability.

Three observations regarding hypnotic suggestibility will be discussed here. First, several studies (McConkey et al., 1980; Woody et al., 2005) have shown that hypnotic suggestibility, as measured by common scales, such as HGSHS-A (Shor and Orne, 1962) and SHSC-C (Weitzenhoffer and Hilgard, 1962), does not consist of a unitary capability, but instead is composed of several factors. In other words, the heterogeneity in responding to different hypnotic and posthypnotic suggestions cannot be attributed simply to the difficulty of items; to the contrary, it seems that different items tap into distinguishable capabilities, and therefore, hypnotic suggestibility is composed of several different suggestibilities (McConkey et al., 1980; Woody et al., 2005). SATH accommodates this observation by assuming that direct-ideomotor suggestions require mainly cognitive simulation in contrast to challenge-ideomotor ones that also require sensory attenuation. Further, due to the change in modality, perceptual suggestions should be separated from motor suggestions. SATH expects that there will be more categories of suggestions, but at least in the standard scales of hypnotic suggestibility, these three categories can be distinguished. As SATH assumes that different cognitive capabilities are required to respond to these different categories, they should be separable in terms of their latent factorial structure. However, as these categories rely on shared cognitive abilities, SATH predicts that the latent factors underlying these categories should be correlated. In a confirmatory factor analysis, Zahedi and Sommer (2022) have shown that SATH’s propositions can successfully model hypnotic suggestibility scores measured by the HGSHS-A.

Considering SATH’s postulation that several top-down processes are involved in responding to suggestions, no single cognitive capability, such as the capability to fantasize, suppress irrelevant information, or inhibit prepotent responses, will suffice to respond to all kinds of suggestions (Parris, 2017; Terhune et al., 2017; Lynn et al., 2019). This postulation can explain the mixed results that exist in the field. For instance, several well-conducted recent neurocognitive studies showed that in no-hypnosis conditions, highly suggestible participants performed better in cognitive tasks when compared to low suggestible ones, which was corroborated by the measured neural correlates (Kirenskaya et al., 2019; Srzich et al., 2019). In contrast, Khodaverdi-Khani and Laurence (2016) showed digit span performance in highly suggestible participants is inferior in comparison to low suggestible participants, but there was no significant difference in an N-back task, revealing inconclusive findings with regard to working memory performance. On the other hand, the results of Dienes et al. (2009) in a large sample ( N=180 ) revealed that there was no correlation between hypnotic susceptibility and cognitive capabilities. Based on SATH, studies investigating the relation between hypnotizability and other processes are not useful unless they take into account the factorial nature of these scales.

Here, it should be once more highlighted that cognitive simulation and sensory attenuation are not complicated and special cognitive processes. For instance, top-down controlled attenuation of sensory input can also be observed in non-human species (Saalmann and Kastner, 2009; Manita et al., 2015). In other words, regardless of baseline cognitive capabilities, to some extent, all participants can exert top-down control over perception. For example, in two previous studies with healthy participants, all of them showed top-down attenuation of neural activity, regardless of their performance in other tasks (Lopresti-Goodman et al., 2013; Fazeli et al., 2014).

The second observation is related to inter-individual differences in hypnotic suggestibility. Previous studies (Terhune and Cardeña, 2010; Terhune et al., 2011; Terhune, 2015) have shown that there are at least two different groups of highly suggestible participants. However, these categorical differences cannot explain the dimensional characteristics of hypnotizability scores discussed above (Reshetnikov and Terhune, 2022). These two groups can be described as being high in dissociative tendencies versus avid users of imaginative capabilities (Pekala et al., 1995; Barrett, 1996; Barber, 1999; Barber, 2000). This observation can be accommodated as well via the propositions of SATH. Even when considering a single hypnotic or posthypnotic suggestion, participants might use different mechanisms to different extents to comply with it. A participant capable of vividly simulating suggested stimuli but less capable of allocating amplified attention to actual sensory input may rely on cognitive simulation to respond to direct verbal suggestions. This participant can render virtual somatosensory prediction errors more precise than predictions by cognitive simulation. Conversely, a person with the opposite distribution of capabilities, *ceteris paribus*, may rely more on sensory attenuation to decrease the precision of somatosensory input. Therefore, SATH predicts that there are different groups of highly suggestible participants who rely mainly on different capabilities to respond to direct verbal suggestions.

The last point is related to psychosocial antecedents that can affect responsiveness to suggestions. These antecedents are precisely what Kirsch (1997) assumes for distinguishing between suggestibility and hypnotic suggestibility, which originated from the works of Hilgard (1965a,b). Accordingly, SATH assumes that, besides cognitive capabilities, psychosocial factors affect suggestibility. Notably, psychosocial factors are of unique importance for determining hypnotizability, that is, the increase in suggestibility due to hypnotic induction (Kirsch, 1997). In line with this claim, it has been shown that when measuring hypnotic suggestibility – the combined effect of suggestibility and hypnotizability – psychosocial factors such as willingness to be hypnotized and openness of participants (Green and Lynn, 2011; Lynn et al., 2015b), expectations about hypnosis (Kirsch and Lynn, 1997), rapport with the hypnotist (Lynn et al., 2019), and motivation to respond to suggestions (Jones and Spanos, 1982) are relevant.

Corroborating this hypothesis, the results of Zahedi and Sommer (2022) suggested that a bifactorial model can explain the variance in hypnotic suggestibility, as measured by HGSHS-A, scores better than normal multifactorial ones. Bifactorial models show that two sources of variance are simultaneously affecting the data (Reise, 2012; Eid et al., 2018). Hence, this result might corroborate the hypothesis of Kirsch (1997) and Hilgard (1965a,b), which is also echoed by SATH.

## 5 Comparison with other theories of hypnosis based on PCF

SATH is not the only or the first hypnosis theory that is based on the PCF. To the best of our knowledge (for a systematic review, see Zahedi et al., 2021), two other hypnosis theories use elements of the PCF, which will be presented shortly here due to length limits. The first is Interoceptive Predictive Coding (Jamieson, 2016; Jamieson, 2021). Jamieson (2016) uses a combination of the PCF and comparator model (Frith et al., 2000), where it is necessary to have two copies of any motor command, one for predicting the consequences of the action and the other for conducting it. To understand the altered SoA and SoR while responding to motor suggestions, Jamieson (2016) argues that the misattribution of movements to external sources in hypnotized participants must be related to the formation of internal models based on the hypnotist’s suggestions. Further, these internal models are not implemented through normal pathways but by lower-level perceptual and proprioceptive units. Therefore, no interoceptive predictions will be formed, and due to their absence, participants cannot recognize the source of their actions. In a new iteration of his theory, Jamieson (2018) further explains that during hypnotic responses, the generative models cannot be updated based on feedback from reality, as doing so will align generative models with reality.

In the second predictive coding model, Martin and Pacherie (2019) proposed that, in contrast to active inference under normal conditions, during responses to hypnotic suggestions, somatosensory and proprioceptive signals are not attenuated but are even more precise compared to normal conditions. However, during hypnosis also, predictions will be more precise compared to normal conditions because they are based on hypnotic suggestions. Critically, during hypnosis, in a fast-altering manner, predictions will be given a higher and then lower weight compared to prediction errors, which provides windows where action can take place. As both predictions and somatosensory feedback are precise, a persistent and unresolved prediction error is generated. To interpret strong prediction error signals during hypnosis, participants will attribute their actions to external forces.

SATH is distinguishable from both of the accounts of Jamieson (2016) and Martin and Pacherie (2019) as it, first, assumes several mechanisms underlying the effects of suggestions in order to explain multiple groups of highly hypnotizable participants and the multifactorial structure of suggestibility. Second, unlike the account of Jamieson (2016), SATH does not assume an altered state of consciousness to explain the effects of suggestions but argues that implementing normal cognitive processes can result in the observed effects. Third, Martin and Pacherie (2019) focus on motor suggestions, and their generalization to other forms of suggestions requires implementing new insights or elements, which are already explicated by SATH. Finally, SATH introduces an expansion of the PCF rather than an exception to its underlying mechanisms, which is clear if one considers the similarity between SATH and other PCF modules, such as the accounts of hysteria by Edwards et al. (2012).

## 6 Conclusion

In the current paper, we proposed a new framework, called SATH, for understanding hypnotic phenomena. SATH is based on the PCF and ambitiously expands it to account for the objective and subjective effects of direct verbal suggestions, including hypnotic and posthypnotic ones. Specifically, *SATH* focuses on three top-down cognitive processes, namely, *(1)* cognitive simulation, *(2)* neural adaptation, and *(3)* learning through simulation. The core postulations of SATH can be summarized as follows: (1) by simulating proprioceptive, interoceptive, and exteroceptive signals, individuals can produce precise predictions that dominate unattenuated prediction errors. (2) The top-down controlled attenuation of sensory prediction errors due to neural adaptation can make sensory feedback from external sources less precise than from simulated ones. Furthermore, (3) through simulations, individuals can learn new stimulus–response or stimulus-outcome associations. Together, these three postulations can mechanistically explain a wide range of objective and subjective effects of hypnotic phenomena. We believe the suggested framework is exhaustive and parsimonious and provides many testable hypotheses about the basic mechanisms involved in responding to direct verbal suggestions, including hypnotic ones. Therefore, in line with criteria outlined by philosophers of science, such as Popper (1971), SATH should be able to advance our understanding of hypnotic phenomena by accounting for many existing findings and providing viable avenues for future research.

## Data availability statement

The original contributions presented in the study are included in the article/supplementary material, further inquiries can be directed to the corresponding author.

## Author contributions

AZ: Conceptualization, Funding acquisition, Resources, Writing – original draft, Writing – review & editing. SL: Writing – review & editing, Supervision. WS: Resources, Supervision, Writing – original draft, Writing – review & editing, Funding acquisition.

## Footnotes

^1^Interestingly, the criteria of parsimony and adequacy (Popper, 1971) apply to active inference accounts of sense-making in the brain. One way of understanding predictive coding is in terms of maximizing the evidence for generative (world) models of the sensorium. The logarithm of this model evidence is equal to accuracy minus complexity. This is congruent with the imperatives for adequacy and parsimony. In other words, self-evidencing (Hohwy, 2014) complies with the same principles as would apply to the brain as a little scientist (Bruineberg et al., 2018). Therefore, by basing SATH on the PCF, it is possible to adhere to Popper’s criteria for a new theory.

^2^Notably, SATH also predicts an initial increased activity in the sensors, the primary somatosensory, or associated brain cortices. However, this increase might happen preemptively and should not happen after the perception of each stimulus. It might also happen during the reception of the suggestion rather than its execution, or be restricted to early trials rather than later ones. To the best of our knowledge, there is no study that corroborates or refutes this hypothesis.

## In memoriam

Unfortunately, our great friend and co-author, Steven Jay Lynn, passed away during the revision of the current paper. However, as he had an immense effect on this contribution and our thinking about hypnosis in general, we decided to maintain him as a co-author on the paper. May this serve as a tribute to his memory.
